# Terpene synthases from *Cannabis sativa*

**DOI:** 10.1371/journal.pone.0173911

**Published:** 2017-03-29

**Authors:** Judith K. Booth, Jonathan E. Page, Jörg Bohlmann

**Affiliations:** 1 Michael Smith Laboratories, University of British Columbia, East Mall, Vancouver, B.C., Canada, V6T 1Z4; 2 Anandia Laboratories, Lower Mall, Vancouver, B.C., Canada, V6T 1Z4; 3 Botany Department, University of British Columbia, University Blvd, Vancouver, B.C., V6T 1Z4; Michigan State University, UNITED STATES

## Abstract

Cannabis (*Cannabis sativa*) plants produce and accumulate a terpene-rich resin in glandular trichomes, which are abundant on the surface of the female inflorescence. Bouquets of different monoterpenes and sesquiterpenes are important components of cannabis resin as they define some of the unique organoleptic properties and may also influence medicinal qualities of different cannabis strains and varieties. Transcriptome analysis of trichomes of the cannabis hemp variety ‘Finola’ revealed sequences of all stages of terpene biosynthesis. Nine cannabis terpene synthases (CsTPS) were identified in subfamilies TPS-a and TPS-b. Functional characterization identified mono- and sesqui-TPS, whose products collectively comprise most of the terpenes of ‘Finola’ resin, including major compounds such as β-myrcene, (*E*)-β-ocimene, (-)-limonene, (+)-α-pinene, β-caryophyllene, and α-humulene. Transcripts associated with terpene biosynthesis are highly expressed in trichomes compared to non-resin producing tissues. Knowledge of the CsTPS gene family may offer opportunities for selection and improvement of terpene profiles of interest in different cannabis strains and varieties.

## Introduction

**Fig 1:**
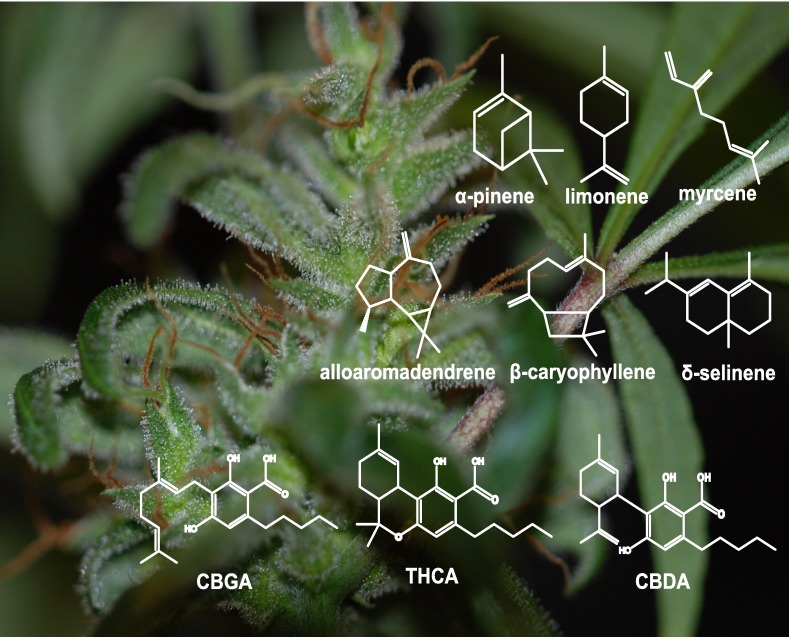
Glandular trichomes on the surface of pistillate inflorescences and leaves of *Cannabis sativa* ‘Finola’. The inflorescence (left) with a high density of glandular trichomes was at five weeks post onset of flowering. Non-inflorescence leaves (right) have lower density of glandular trichomes. Structures of representative cannabis resin components are shown in white: monoterpenes (top row), sesquiterpenes (middle row), and cannabinoids (bottom row). GBGA = cannabigerolic acid; THCA = tetrahydrocannabinolic acid; CBDA = cannabidiolic acid.

*Cannabis sativa*, referred to here as cannabis, has been used for millennia as a medicine and recreational intoxicant [1, 2]. The species *Cannabis sativa* comprises both marijuana and hemp [3, 4, 5]. Medicinal cannabis is highly valued for its pharmacologically active cannabinoids, a class of terpenophenolic metabolites unique to cannabis. These compounds are primarily found in the resin produced in the glandular trichomes of pistillate (female) inflorescences. Cannabis resin also contains a variety of monoterpenes and sesquiterpenes (Fig 1), which are responsible for much of the scent of cannabis flowers and contribute characteristically to the unique flavor qualities of cannabis products. Similarly, terpenes in hop (*Humulus lupulus*), a close relative of cannabis, are an important flavoring component in the brewing industry. Differences between the pharmaceutical properties of different cannabis strains have been attributed to interactions (or an ‘entourage effect’) between cannabinoids and terpenes [6, 7]. For example, the sesquiterpene β-caryophyllene interacts with mammalian cannabinoid receptors [8]. As a result, medicinal compositions have been proposed to incorporate blends of cannabinoids and terpenes [9]. Terpenes may contribute anxiolytic, antibacterial, anti-inflammatory, and sedative effects [6].

**Fig 2:**
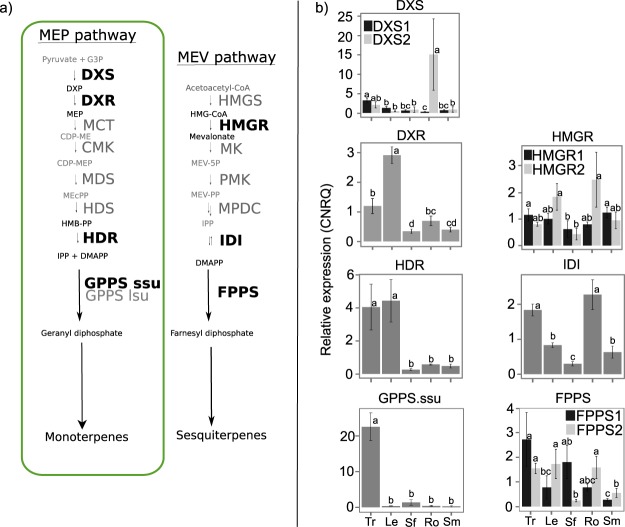
Schematic of the plastidial methylerythritol phosphate pathway (MEP) and mevalonic acid pathway (MEV) and transcript abundance in different parts of cannabis. Steps shown in bold (a) were included in the qPCR analysis (b) of relative abundance of transcripts. Letters indicate significantly different means between tissues (tested within each gene), Fisher’s LSD (alpha = 0.05). Abbreviations: Tr = trichome; Le = leaf; Sf = stamenate flower; Ro = root; Sm = stem.

Terpene biosynthesis in plants involves two pathways to produce the general 5-carbon isoprenoid diphosphate precursors of all terpenes, the plastidial methylerythritol phosphate (MEP) pathway and the cytosolic mevalonate (MEV) pathway. These pathways ultimately control the different substrate pools available for terpene synthases (TPS). The MEP pathway is comprised of seven steps that convert pyruvate and glyceraldehyde-3-phosphate into isopentenyl diphosphate (IPP) and dimethylallyl diphosphate (DMAPP) (Fig 2A). Enzymes thought to be critical for flux regulation through this pathway include the first two and final two steps: 1-deoxy-D-xylulose 5-phosphate synthase, 1-deoxy-D-xylulose 5-phosphate reductase, 4-hydroxy-3-methylbut-2-enyl diphosphate synthase, and 4-hydroxy-3-methylbut-2-enyl diphosphate reductase [10, 11]. The MEV pathway converts three units of acetyl-CoA to IPP, which is then isomerized to DMAPP by IPP isomerase. A rate-limiting step in this six-step pathway is 3-hydroxy-3-methylglutaryl-CoA reductase, which produces mevalonate [12]. IPP and DMAPP are condensed into longer-chain isoprenoid diphosphates by prenyltransferases, which include geranyl diphosphate (GPP) synthase (GPPS) and farnesyl diphosphate (FPP) synthase (FPPS). GPPS and FPPS condense one unit of IPP and one or two units of DMAPP to form 10- and 15-carbon linear *trans*-isoprenoid diphosphates, respectively. GPP is the 10-carbon precursor of monoterpenes and is typically derived from 5-carbon isoprenoid diphosphate units of the MEP pathway. GPP is also a building block in the biosynthesis of cannabinoids [13, 14]. FPP is the 15-carbon precursor of sesquiterpenes and is commonly produced from 5-carbon isoprenoid diphosphate units of the cytosolic mevalonate (MEV) pathway. GPPSs exist as homo- or heterodimeric enzymes. In hops, the closest known relative of cannabis, heterodimeric GPPSs can produce both GPP and the 20-carbon geranylgeranyl diphosphate (GGPP), with the ratio of large to small G(G)PPS subunits controlling the product outcome [15, 16, 17]. The linear isoprenoid diphosphates GPP and FPP are substrates for monoterpene synthases (mono-TPS) and sesquiterpene synthases (sesqui-TPS), respectively, which diversify these precursors into a large number of different mono- and sesquiterpenes.

TPS are typically encoded in large and diverse gene families in plants [18], where they contribute to both general and specialized metabolism. The plant TPS gene family has been annotated with six subfamilies. In angiosperms, the subfamily TPS-b is typically comprised of mono-TPS and TPS-a enzymes are often sesquiterpene synthases. TPS produce cyclic and acyclic terpenes via carbocationic intermediates, formed by divalent metal co-factor dependent elimination of the diphosphate. The reactive cationic intermediate can undergo cyclization and rearrangements until the reaction is quenched by deprotonation or water capture [19]. Many TPS form multiple products from the same substrate.

The terpene composition of cannabis resin varies substantially based on genetic, environmental, and developmental factors [20, 21, 22, 23]. Concentrations and ratios of cannabinoids are relatively predictable for different strains, but terpene profiles are often unknown or unpredictable [20, 23]. To select and improve cannabis strains with desirable terpene profiles, it is necessary to identify genes responsible for terpene biosynthesis, which can be accomplished by harnessing available cannabis transcriptome and genome resources. Draft genomes and transcriptomes for the marijuana strain Purple Kush and the hemp variety ‘Finola’ have previously been published [24]. We used these resources to explore the expression of genes involved in all stages of terpene biosynthesis. We identified nine *TPS* gene models in the ‘Finola’ transcriptome. *TPS* genes and gene transcripts in the MEP and MEV pathways were highly expressed in floral trichomes. We identified biochemical functions of *TPS* that are highly expressed in ‘Finola’. The TPS enzymes characterized account for most of the terpenes found in ‘Finola’ resin.

## Materials and methods

### Plant materials

Cannabis seeds, ‘Finola’, were obtained from Alberta Innovates Technology Futures (www.albertatechfutures.ca). All plants were grown indoors in a growth chamber under a Health Canada license. Seeds were germinated on filter paper, then transferred to 4:1 Sunshine Mix #4 (www.Sungro.com):perlite. Daylight length was 16 h under fluorescent lights, and ambient temperature 28°C. About two weeks after germination, seedlings were transferred to larger pots. After repotting, all plants were fertilized weekly with Miracle-Gro all-purpose plant food (24-8-26) (www.miraclegro.com) according to manufacturer’s instructions.

### Terpene extraction

Pistillate inflorescences were collected and trimmed of leaves and stems. All flowers from an individual plant were pooled. Tissue samples of ~0.2 g were weighed to determine fresh weight. Three rounds of extraction in 1 ml of pentane were performed for 1 hour each at room temperature with gentle shaking. Isobutyl benzene was added as an internal standard. After three extractions, no terpenes were identified in a fourth solvent extraction. Floral tissue was then dried overnight and weighed to determine dry weight. All three pentane extracts were combined for a total volume of 3 ml for analysis.

### Trichome isolation

The heads of glandular trichomes were isolated from whole inflorescences as previously described [25] without XAD-4 and with the addition of 5 mM aurintricarboxilic acid in the isolation buffer. Instead of a cell disruptor, floral tissue was vortexed with glass beads in a Falcon tube to remove trichome heads. After vortexing, trichomes were separated from beads and green tissue by filtration through a 105 μm nylon mesh. Trichomes were concentrated by gentle centrifugation in ice-cold buffer. The supernatant was removed with a pipette, and the pellet of trichomes was immediately frozen in liquid nitrogen.

### Metabolite analysis

Gas chromatography (GC) analysis of floral extracts was performed on an Agilent (www.chem.agilent.com) 7890A GC with a 7683B series autosampler and 7000A TripleQuad mass spectrometer (MS) detector at 70 eV electrospray ionization with a flow rate of 1 ml min^-1^ He. The column was an Agilent VF-5MS or DB-5MS (30 m, 250 μm internal diameter, 0.25 μm film). The following temperature program was used: 50°C, then increase 150°C min^-1^ to 320°C, hold for 5 minutes. Injection was pulsed splitless at 250°C. Compounds were identified by comparison of retention index and mass spectra to authentic standards. Standards were available for all monoterpenes and the following sesquiterpenes: β-caryophyllene, α-humulene, farnesol, valencene, germacrene D, and alloaromadendrene. Tentative identifications for all other sesquiterpenes were made by comparison of retention index and mass spectra to National Institute of Standards and Technology (NIST) MS library. Identifications of bergamotene, δ-selinene, and farnesene were strengthened by comparison to essential oils of *Citrus bergamia* (Bergamot) and *Pimenta racemose* (Bay) (www.lgbotanicals.com). TPS assay products were analyzed by the same procedure described above for plant extracts, but with the following temperature program: 50°C for 3 minutes, then increase 15°C min^-1^ to 280°C, hold for 2 minutes. Assay products were analyzed using Agilent HP-5 and DB-Wax columns (30 m length, 250 μm internal diameter, 0.25 μm film). For cold injection of sesqui-TPS assay products, the following program was used on a DB-Wax column: 40°C for 3 minutes, then increase 10°C min^-1^ to 230°C, hold for 7 minutes. Injection was at 40°C with a 1:1 split ratio. Chiral analysis of terpenes was done using a Cyclodex-B column (30 m length, 250 μm internal diameter, 0.25 μm film). Injection was pulsed splitless, with the following program: 40°C for 1 minute, then increase 5°C min^-1^ to 100°C, then increase 15°C min^-1^ to 250°C, hold for 4 minutes. Chirality was determined by retention index and comparison with authentic standards.

### cDNA cloning and characterization of *TPS* genes

Total RNA was isolated from ‘Finola’ flowers, leaves, stem, and roots using Invitrogen PureLink Plant RNA reagent (www.thermofisher.com). RNA quality and concentration was measured with a Bioanalyzer 2100 RNA Nanochip assay (www.agilent.ca). cDNA was synthesized with the Superscript III reverse transcriptase kit (Thermo Fisher). Full length and N-terminally truncated cDNAs without transit peptides where applicable [26] were amplified from cDNA using gene-specific primers (S1 Table) designed from published transcriptomic data [24]. N-terminal transit peptides were predicted based on sequence alignments [27] and using the TargetP and ChloroP servers [28]. PCR amplified ‘Finola’ cDNAs were ligated into pJET vector (www.clontech.com) for sequence verification, and subcloned into expression vectors pET28b+ (www.endmillipore.ca) or pASK-IBA37 (www.iba-lifesciences.org) in the case of CsTPS5FN.

High-confidence full-length *TPS* cDNA candidates from Purple Kush (CsTPS13PK, CsTPS30PK, and CsTPS33PK) were synthesized by GenScript (www.genscript.com) into pET28b+. For this purpose, putative *TPS* sequences from Purple Kush transcriptome data were verified by comparison to genomic sequences [24].

Plasmids were transformed into *E*. *coli* strain BL21DE3-C43 for heterologous protein expression, as previously described [29]. Recombinant protein was purified using the GE healthcare His SpinTrap kit (www.gelifesciences.com) according to manufacturer’s instructions. Binding buffer for purification was 20 mM 2-[4-(2-hydroxyethyl)piperazin-1-yl]ethanesulfonic acid (HEPES) (pH 7.5), 500 mM NaCl, 25 mM imidazole, and 5% glycerol. Cells were lysed in binding buffer supplemented with Roche complete protease inhibitor tablets (lifescience.roche.com) and 0.1 mg ml^-1^ lysozyme. Elution buffer was 20 mM HEPES (pH 7.5), 500 mM NaCl, 350 mM imidazole, and 5% glycerol. Purified protein was desalted through Sephadex into TPS assay buffer. *In vitro* assays were performed in 500 μl volume by incubating purified protein with isoprenoid diphosphate substrates (Sigma) as previously described [30], except that the TPS assay buffer was 25 mM HEPES (pH 7.3), 100 mM KCl, 10 mM MgCl_2_, 5% glycerol, and 5 mM DTT. Isoprenoid diphosphate substrates were dissolved in 50% methanol and added to the assay at final concentrations of 32 μM (GPP) and 26 μM (FPP). Enzyme concentrations were variable ranging from 20 to 100 μg per 500 μl assay volume. Assays were overlaid with 400 μl hexane or pentane, with 2.5 μM isobutyl benzene as internal standard.

### *Nicotiana benthamiana* transformation and transient expression

The CsTPS5FN coding sequence was inserted into the Golden Gate plant expression vector pEAQ-GG, which contains a CaMV 35S promoter. This construct and the suppressor-of-silencing gene p19 were transformed into *Agrobacterium tumefasciens* strain AGL1. For infiltration, *A*. *tumefasciens* was grown overnight as previously described [31], then pelleted and resuspended in 10 mM 2-(N-morpholine)-ethanesulphonic acid (MES) buffer, pH 5.8, 10 mM MgCl_2_, 20 μM acetosyringone to OD_600_ 0.5. Equal volumes of bacteria, 25 ml each, containing TPS5 and p19 were infiltrated into the abaxial side of 4-week-old *N*. *benthamiana* plants. Infiltrated plants were grown for three days in the dark. Infiltrated leaves were harvested and ground in TPS assay buffer, and enzyme activity assays were conducted as above.

### RT-qPCR analysis of transcript abundance

cDNA for qPCR was synthesized using the Maxima First Strand cDNA synthesis kit (Thermo Fisher) according to manufacturer’s instructions. qPCR reactions were done in 15 μl volumes with SsoFast EvaGreen supermix (Bio-Rad), 4 μl template (2 ng), and 0.3 μM primers. Primers (S2 Table) were designed using Primer3 software [32]. Reference genes were chosen by geNorm [33], analyzed with qBase+ software (www.biogazelle.com). Reference genes used for RT-qPCR of early isoprenoid biosynthesis across different plant organs were *actin* and *CDK3*. For RT-qPCR of *TPS* transcripts in trichomes, reference genes were *CDK3* and *GAPDH*. RT-qPCR analyses were done with four biological and two technical replicates for the early isoprenoid biosynthetic transcripts in different organs. For *TPS* transcript analysis in trichomes, three biological and three technical replicates were performed. Gene expression was analyzed using qBase+. Statistical analysis was performed by ANOVA on log-transcript abundance, with Bonferroni correction.

### *TPS* gene prediction and phylogeny

‘Finola’ genome and transcriptome assemblies [24] were downloaded from the cannabis genome browser (http://genome.ccbr.utoronto.ca/cgi-bin/hgGateway). These assemblies were used as the subject of a tBLASTn search using 71 *TPS* genes (S3 Table) downloaded from GenBank and Phytozome. Gene and splice site prediction was performed on scaffolds containing regions with similarity to *TPS* sequences using the Exonerate gene prediction algorithm [34]. A preliminary Purple Kush genome assembly based on PacBio (www.pacb.com) sequencing data was also used. Predicted genes were manually curated against earlier Purple Kush sequence data, and examined to establish open reading frames, start codons, and stop codons. A maximum likelihood phylogeny was built using phylogeny.fr [35]. The alignment used for input was built using the MUSCLE algorithm with all translated amino acid sequences from the predicted *TPS* gene models from cannabis and the 71 published *TPS* sequences listed above. Alignments were curated using the Gblocks algorithm, and tree construction was performed using PhyML 3.0 with 100 bootstrap replicates.

## Results

### Terpene profiles of cannabis inflorescences

We used the *C*. *sativa* oilseed hemp variety ‘Finola’ to investigate terpene profiles of pistillate flowers. ‘Finola’ was chosen because reference draft genome and transcriptome assemblies were published [24]. Pistillate flowers, which have the highest density of glandular trichomes relative to other parts of the plant (Fig 1), were sampled to cover early to mid-stage inflorescences between three and eight weeks post onset of flowering, where onset of flowering is defined as the first appearance of pistils. Independent of the stage of inflorescence, the most abundant monoterpenes were myrcene, (+)-α-pinene, (-)-limonene, (+)-β-pinene, terpinolene, and (*E*)-β-ocimene (Table 1). The most abundant sesquiterpenes were β-caryophyllene, α-humulene, bergamotene, and farnesene. Terpene profiles showed considerable variations between individual plants as indicated with the relatively high standard deviation (Table 1). No trends were observed for individual metabolites as a function of inflorescence development, but total monoterpenes increased compared to sesquiterpenes as inflorescences matured. Mid-stage flowers (~4 weeks post onset of flowering) had a mean monoterpene content of 389 μg g^1^ DW (SE = 44, n = 9), and a mean sesquiterpene content of 34 μg g^1^ DW (SE = 6.3, n = 9).

**Table 1:** Relative composition of terpene profiles in *C*. *sativa* 'Finola' pistillate flowers. Twenty two individual plants were sampled. Contribution of individual terpenes is expressed as a proportion of the total terpenes within a given class (i.e., monoterpenes or sesquiterpenes).

**Metabolite**	**Percent Proportion (mean ± st. dev)**	**Terpene class**
**(+)-α-Pinene**	23 ± 17	Monoterpene
**(+)-β-Pinene**	8.6 ± 4.6	Monoterpene
**Myrcene**	27 ± 21	Monoterpene
**(-)-Limonene**	12 ± 10	Monoterpene
**(*E*)-β-Ocimene**	10 ± 6.5	Monoterpene
**Terpinolene**	18 ± 14	Monoterpene
**β-Caryophyllene**	46 ± 13	Sesquiterpene
**Bergamotene**	3.6 ± 3.0	Sesquiterpene
**Farnesene**	4.4 ± 3.6	Sesquiterpene
**α-Humulene**	19 ± 7.6	Sesquiterpene

### Transcriptome mining of early isoprenoid biosynthesis genes

We queried the ‘Finola’ transcriptome for transcripts involved in the early stages of isoprenoid biosynthesis. We combined four transcriptome sets downloaded from the Cannabis Genome Browser (http://genome.ccbr.utoronto.ca/cgi-bin/hgGateway), including transcripts from developing seeds, mature pistillate flowers, stamenate (male) flowers, and whole seedlings. The tBLASTn algorithm was used to search translated ‘Finola’ nucleotide sequences, using amino acid sequences from *Vitis vinifera* and *Arabidopsis thaliana*, and an e-value cut-off of 1^−10^.

At least one full-length or nearly full-length (>95%) transcript was found for each of the core genes in the MEP and MEV pathways, and linear isoprenoid diphosphate prenyltransferases (Fig 2B). The genes included in the analysis of the MEP pathway were 1-deoxy-D-xylulose 6-phosphate (DOXP) synthase (DXS), DOXP reductoisomerase (DXR), 2-C-methyl-D-erythritol cytidyltransferase (MCT), 4-diphosphocytidyl-2-C-methyl-D-erythritol kinase (CMK), 4-hydroxy-3-methyl-but-2-enyl diphosphate (HMB-PP) synthase (HDS), and HMB-PP reductase (HDR). Two versions of DXS, CsDXS1 and CsDXS2, were found, which are 62.8% identical at the amino acid level. In a phylogeny, CsDXS1 clusters with members of the DXS subfamily DXS-I of other plant species, and CsDXS2 clusters with members of the DXS-II subfamily (S1 Fig).

The genes included in the MEV pathway analysis were 3-hydroxy-3-methylglutaryl-CoA (HMG-CoA) synthase (HMGS), HMG-CoA reductase (HMGR), mevalonate kinase (MK), phospho-mevalonate kinase (PMK), mevalonate-5-phosphate decarboxylase (MPDC), and IPP isomerase (IDI). At least one transcript was found corresponding to each enzyme. Two transcripts were found for HMGR, HMGR1 and HMGR2, which are 72.7% identical at the amino acid level.

As candidate prenyltransferases, we found transcripts of a heterodimeric GPPS system similar to that characterized in hop [17], with a GPPS large subunit (GPPS.lsu) and a GPPS small subunit (GPPS.ssu). Two transcripts were identified corresponding to FPPS, 80.3% identical to one another at the amino acid level.

### RT-qPCR expression analysis of isoprenoid biosynthetic transcripts

To assess gene expression of isoprenoid biosynthesis across different parts of the cannabis plant, we used qRT-PCR to examine the transcript abundance of prenyltransferases and key genes in the MEP and MEV pathways. We selected genes for three steps in the MEP pathway, *DXS*, *DXR*, and *HDR*, as well as two MEV pathway genes, *HMGR* and *IDI*. We also included the two *FPPS* genes. In heterodimeric GPPS, the rate of GPP biosynthesis is governed by ratios of small to large subunits, with higher ratios of small to large subunits leading to higher GPP formation [17]. We therefore measured transcript levels of the *GPPS*.*ssu* gene. Transcript levels were determined in ‘Finola’ leaves, stems, roots, staminate flowers, and glandular trichomes from pistillate flowers. Pistillate flowers were harvested between 10–12 weeks post germination.

*CsDXS1* was expressed in all samples, with no significant differences between different parts of the plant (Fig 2B). *CsDXS2* was also expressed in all samples. Levels of *CsDXS1* and *CsDXS2* transcripts were not significantly different except in roots, where average *CsDXS2* levels were 14-fold more abundant than *CsDXS1*. *HMGR1* and *HMGR2* were both expressed in every sample. Their transcript abundances were not significantly different, except in leaves and roots where *HMGR2* was significantly more highly expressed. Abundances of *FPPS* transcripts in roots and staminate flowers were significantly different between the two *FPPS* genes. The MEP pathway genes *DXR* and *HDR* were significantly more highly expressed in trichomes and leaves. Cannabis leaves bear glandular trichomes, but much less densely than flowers. Genes in the MEP pathway were also more highly expressed in trichomes and leaves than genes of the MEV pathway. Transcripts of *GPPS*.*ssu* were very highly abundant (>25 fold higher) in trichomes compared to other tissues.

### Members of the cannabis *TPS* gene family

**Fig 3:**
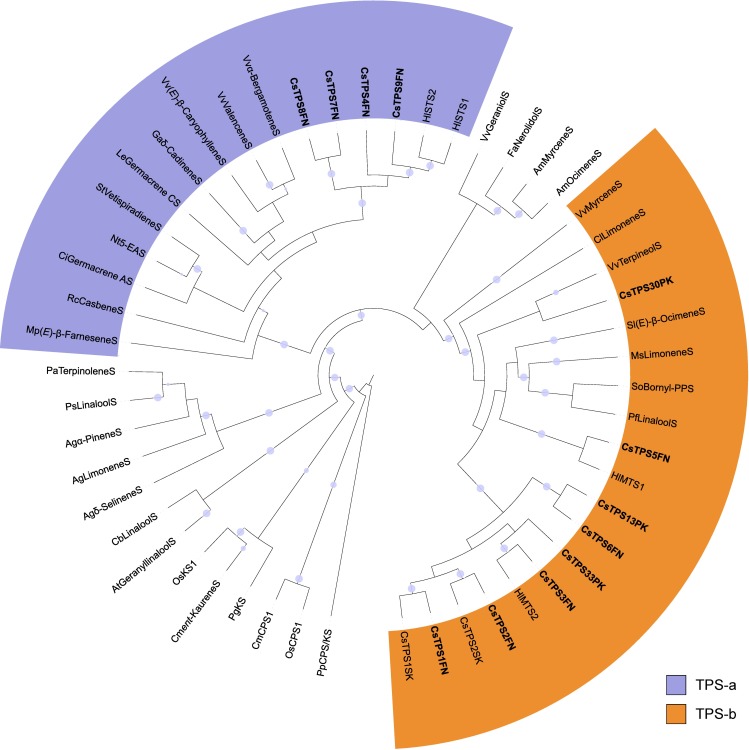
Maximum likelihood phylogeny of CsTPS. Within the TPS-a and TPS-b subfamilies, TPS from the *Cannabaceae*, including cannabis and hops, are more closely related to one another than to TPS from other angiosperms. Cannabis TPS are in bold. The cannabis strain or variety of origin is indicated by two letters following the TPS#: FN: ‘Finola’, SK: ‘Skunk’, PK: Purple Kush. Branches with bootstrap values >80% (100 repetitions) are indicated with a grey dot. TPS of other species included are from Pp: *Physcomitrella patens*, Os: *Oryza sativa*, Cm: *Cucurbita maxima*, At: *Arabidopsis thaliana*, Cb: *Clarkia breweri*, Ag: *Abies grandis*, Pa: *Picea abies*, Fa: *Fragaria ananassa*, Am: *Antirrhinum majus*, Mp: *Mentha x piperita*, Rc: *Ricinus communis*, Ci: *Cichorium intybus*, Sl: *Solanum lycopersicum*, Nt: *Nicotiana tabacum*, Le: *Lycopersicum esculentum*, Ga: *Gossypium arboreum*, St: *Solanum tuberosum*, Vv: *Vitis vinifera*, Hl: *Humulus lupulus*, So: *Salvia officinalis*, Cl: *Citrus limon*, Ms: *Mentha spicata*, Pf: *Perilla frutescens*. ‘S’ suffix = synthase.

In the ‘Finola’ (FN) trichome transcriptome we identified nine full-length or nearly full-length (predicted >95% of amino acid length) and six partial putative *TPS* genes (*CsTPS FN*). A maximum likelihood phylogeny of the nine full-length CsTPSFN predicted protein sequences and representative TPS from other plant species placed the CsTPSFN most closely with each other and with TPS from hop (HlSTS1 and HlSTS2) indicating a recent expansion of *TPS* genes in the *Cannabaceae* (Fig 3). Five of the nine CsTPSFN (CsTPS1FN, CsTPS2FN, CsTPS3FN, CsTPS5FN, CsTPS6FN) clustered with members of the TPS-b subfamily, and the remaining four (CsTPS4FN, CsTPS7FN, CsTPS8FN, CsTPS9FN) clustered with the TPS-a subfamily. Two of the CsTPSFN TPS-b genes, CsTPS1FN and CsTPS2FN, encode predicted proteins that were 98.7% and 96.8% identical to CsTPS1 and CsTPS2 previously reported [36] and identified there as (-)-limonene synthase (CsTPS1) and (+)-α-pinene synthase (CsTPS2) from the *C*. *sativa* strain ‘Skunk’.

### Functional characterization of *CsTPSFN* TPS-b subfamily members

**Fig 4:**
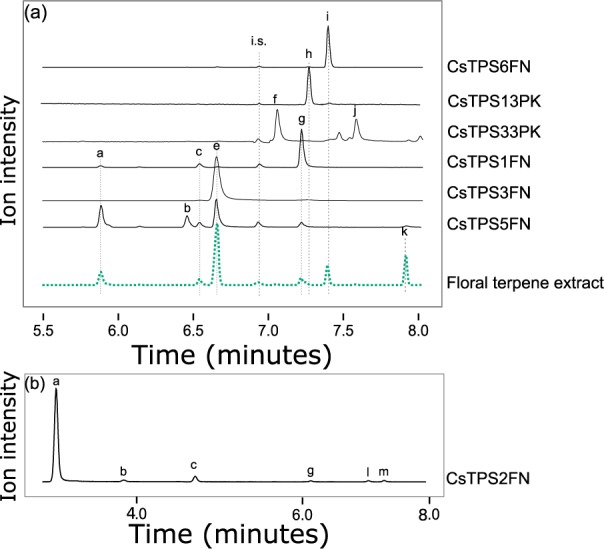
Representative GC-MS traces showing products of *CsTPSFN* TPS-b subfamily members. Black traces show GC-MS total ion chromatogram from CsTPS assays with GPP. Green trace, dotted line, is a representative terpene profile from a `Finola’ inflorescence. (a) shows representative chromatograms from six TPS and a ‘Finola’ floral extract run on an HP-5 GC column. (b) shows the representative chromatogram from CsTPS2FN run on a DB-Wax GC column. Peaks: a) α-pinene, b) camphene, c) sabinene, d) β-pinene, e) myrcene, f) α-terpinene, g) limonene, h) (*Z*)- β-ocimene, i) (*E*)-β-ocimene, j) γ-terpinene, k) terpinolene, l) β -phellandrene, m) isoterpinolene. i.s. = internal standard

*CsTPSFN* were cloned as cDNAs from ‘Finola’ pistillate flowers or synthesized for heterologous expression and identification of product profiles of the encoded enzymes. We cloned four TPS-b family members, *CsTPS1FN*, *CsTPS2FN*, *CsTPS5FN*, and *CsTPS6FN*, from cDNA. *CsTPS3FN* could not be cloned from cDNA and was obtained as a synthetic cDNA. Three TPS-b sequences from the Purple Kush (PK) trichome transcriptome, *CsTPS13PK*, *CsTPS30PK*, and *CsTPS33PK*, were also synthesized for comparison. These five CsTPS from ‘Finola’ and three from Purple Kush were expressed as recombinant proteins and then tested for activity with GPP and FPP and products identified by GC-MS analysis (Fig 4, Table 2, S2 and S3 Figs).

**Table 2:** Functionally characterized CsTPS enzymes.

**Functional gene ID**	**Nearest 'PK' *TPS* gene model**	**Major products**	**Strain of origin**	**Activity on GPP**	**Activity on FPP**
**CsTPS1FN**	CsTPS1PK	(-)-limonene	Finola	+++	np
**CsTPS1SK^ŧ^**	CsTPS1PK	(-)-limonene	Skunk	nd	nd
**CsTPS2FN**	CsTPS2PK	(+)-α-pinene	Finola	+++	np
**CsTPS2SK^ŧ^**	CsTPS2PK	(+)-α-pinene	Skunk	nd	nd
**CsTPS3FN**	CsTPS3PK	β-myrcene	Finola	+++	np
**CsTPS4FN**	CsTPS9PK	alloaromadendrene	Finola	+	+++
**CsTPS5FN**	CsTPS5PK	β-myrcene, (-)-α-pinene	Finola	+++	+
**CsTPS30PK**	CsTPS30PK	β-myrcene	Purple Kush	+++	+
**CsTPS6FN**	CsTPS6PK	(*E*)-β-ocimene	Finola	+++	np
**CsTPS7FN**	CsTPS7PK	δ-selinene*	Finola	+	+++
**CsTPS8FN**	CsTPS8PK	γ-eudesmol*, valencene	Finola	+	+++
**CsTPS9FN**	CsTPS9PK	β-caryophyllene, α-humulene	Finola	+	+++
**CsTPS13PK**	CsTPS13PK	(Z)-β-ocimene	Purple Kush	+++	+
**CsTPS33PK**	CsTPS33PK	α-terpinene, γ-terpinene	Purple Kush	+++	np

^ŧ ^Published in Gunnewich et al., 2008

*Product not compared to authentic standard.

np, no product detected. nd, no data available.

The major product of CsTPS1FN was (-)-limonene, with minor products of (+)-α-pinene, camphene, (+)-β-pinene, and myrcene (Fig 4). CsTPS2FN produced mostly (+)-α-pinene, with minor amounts of (+)-β-pinene, myrcene, (-)-limonene, β-phellandrene and a monoterpene tentatively identified as isoterpinolene (Fig 4). CsTPS3FN produced myrcene as a single detectable product when incubated with GPP (Fig 4). CsTPS30PK also produced only myrcene when tested with GPP (S2 Fig). These two single-product myrcene synthases share only 54.5% amino acid identity. CsTPS5FN also produced myrcene as its most abundant monoterpene product (37%) (Fig 4), but unlike CsTPS3FN and CsTPS30PK, CsTPS5FN produced four additional monoterpenes (-)-α-pinene (23%), (-)-limonene (17%), sabinene (15%), and (-)-β-pinene (8%). The same product profile was identified when CsTPS5FN was transiently expressed in *N*. *benthamiana* (S4 Fig). CsTPS5FN was somewhat unusual among TPS-b members in lacking any obvious N-terminal plastidial targeting sequence. CsTPS5FN also produced minor amounts of farnesene when incubated with FPP, making it the only member of the TPS-b subfamily to produce detectable sesquiterpenes. CsTPS6FN produced 97% (*E*)-β-ocimene with GPP, and the remaining 3% of product was (*Z*)-β-ocimene. A TPS sequence found in Purple Kush, CsTPS13PK, shares 95.5% amino acid sequence identity with CsTPS6FN. CsTPS13PK produces 94% (*Z*)-β-ocimene. A third TPS from Purple Kush, CsTPS33PK, produced two different monoterpenes, α-terpinene (61%) and γ-terpinene (39%) (Fig 4).

### Functional characterization of *CsTPSFN* TPS-a subfamily members

**Fig 5:**
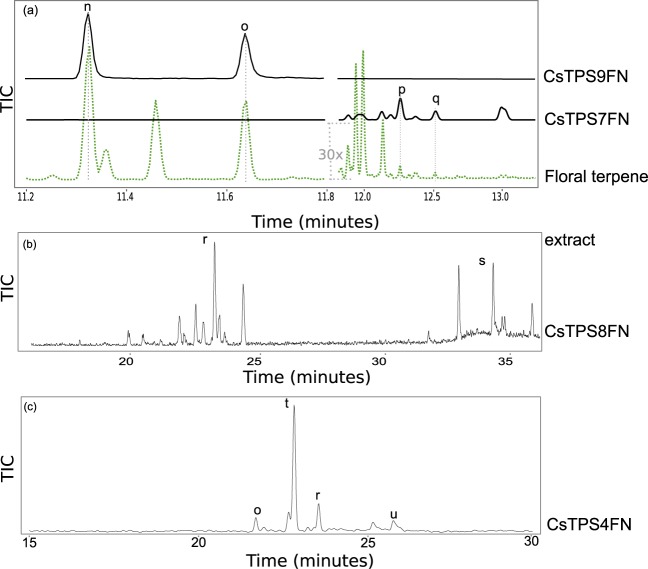
Representative GC-MS traces showing products of *CsTPSFN* TPS-a subfamily members. Black traces show GC-MS total ion chromatogram (TIC) from CsTPS assays with FPP. Green trace, dotted line, in (a) is representative terpene profiles from `Finola’ inflorescences. The right-hand region of the ‘Finola’ terpene profile has been amplified 30-fold to facilitate comparison with the products of CsTPS7FN. (b) shows the trace of CsTPS8FN after cold injection (40°C inlet) onto a DB-wax column. (c) shows the trace for CsTPS4FN on an HP-5 column. Peaks: n) β-caryophyllene, o) α-humulene, p) δ-selinene, q) selina-6-en-4-ol, r) valencene, s) γ-eudesmol, t) alloaromadendrene, u) palustrol.

Four TPS-a family members cloned as cDNAs from ‘Finola’, *CsTPS4FN*, *CsTPS7FN*, *CsTPS8FN*, and *CsTPS9FN*, were expressed as recombinant proteins, proteins tested with GPP and FPP and products identified by GC-MS (Fig 5A, Table 2). CsTPS4FN produced mostly alloaromadendrene (52.3% of total products) with FPP (Fig 5C). The remaining products are a mixture of five sesquiterpene olefins and two alcohols, including valencene, α-humulene, and a product tentatively identified as palustrol. CsTPS4FN was also active with GPP, producing minor amounts of myrcene (S5 Fig). CsTPS7FN produced 21 sesquiterpene olefins and two sesquiterpene alcohols. Of these, products tentatively identified as δ-selinene and selina-6-en-4-ol make up 20.5% and 13.9% of the product profile, respectively. Very few of ‘Finola’ individuals tested contained minor amounts of the products of CsTPS7FN (Fig 5A). The remaining minor products each make up <10% of total sesquiterpene products. When incubated with GPP, CsTPS7FN produced myrcene and limonene (S5 Fig). The most abundant product of CsTPS8FN was initially identified as β-elemol (S6 Fig), which is often an artifact of heat-induced rearrangement. Using a lower injection temperature of 40°C, the β-elemol product was no longer detected and was replaced by peaks corresponding to 11 sesquiterpene olefins and three sesquiterpene alcohols. Of these, two of the major products were identified as γ-eudesmol (19.8%) and valencene (19.6%) (Fig 5B). When CsTPS8FN was incubated with GPP limonene and myrcene were detected (S5 Fig).

CsTPS9FN produced β-caryophyllene and α-humulene from FPP (Fig 5A). These two terpenes are always the most abundant sesquiterpenes in cannabis resin terpene profiles. The CsTPS9FN enzyme produces these two sesquiterpenes in a ratio of approximately 2.5 to 1, which is similar to the ratio of 2.4 +/- 0.2 to 1 observed in ‘Finola’ terpene profiles.

### *CsTPS* transcripts are highly abundant in pistillate inflorescences

To determine to what extent the Cs*TPS* genes described above contribute to the trichome terpene profile, we performed RT-qPCR on transcripts of five *CsTPS* in glandular trichomes isolated from pistillate flowers. Transcript levels of *CsTPS1FN*, *CsTPS2FN*, *CsTPS3FN*, *CsTPS6FN*, and *CsTPS9FN* were examined in trichomes (S7 Fig) isolated from eight ‘Finola’ individuals between two and four weeks post onset of flowering. These five *CsTPS* were chosen because they have a single or at most two products, thus it was deemed more likely to be possible to attempt correlating metabolite abundance with transcript abundances than would be possible with the multiproduct *CsTPS*.

**Fig 6:**
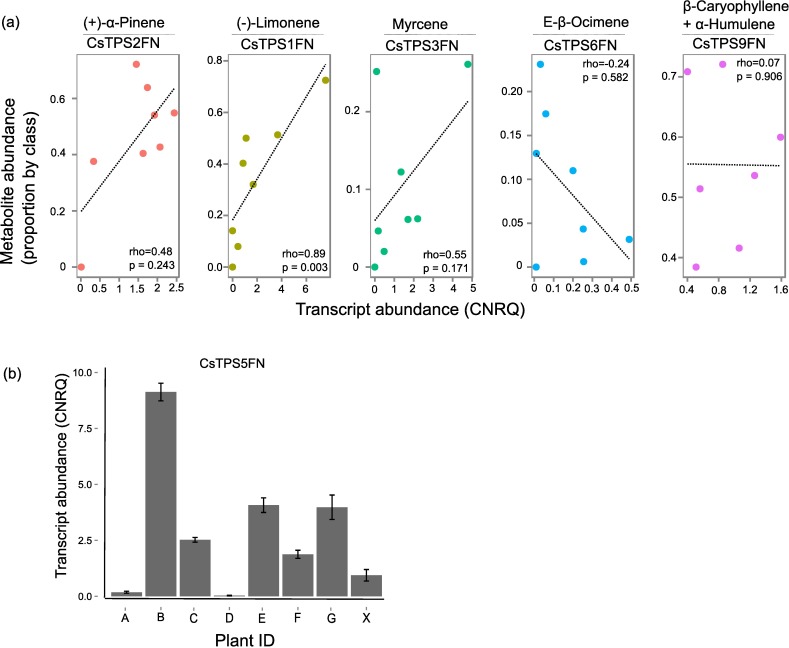
Correlation analysis of metabolite abundance in inflorescence and transcript abundance for five CsTPS in isolated trichomes. (a) Data are shown for five CsTPS/metabolite pairs each in eight ‘Finola’ individuals. Metabolites given with their relative abundance were those that match the product of the corresponding CsTPS. Plant ‘X’ was not included in the left-most panel. Metabolite abundances are expressed as a proportion of the total mono- or sesquiterpenes for each individual. Transcript abundances are calibrated normalized values compared to two reference genes. rho = Spearman rank correlation between transcript and metabolite abundances, p value indicates significance. (b) Transcript abundance of CsTPS5FN in eight ‘Finola’ individuals.

Of the eight individual plants, seven showed typical inflorescence terpene metabolite profiles. Surprisingly, one individual had no detectable inflorescence monoterpenes except for traces of (*E*)-β-ocimene, although it did contain cannabinoids and sesquiterpenes in floral trichomes (S8 Fig). In Fig 6A, metabolite levels of the target compounds are expressed as a proportion of the total mono- or sesquiterpenes in each floral terpene extract. *CsTPS2FN* was the most abundant of the six different *TPS* transcripts measured, and its major product, (+)-α-pinene, was also the most abundant monoterpene on average in the eight plants examined. Similarly, (*E*)-β-ocimene and the (*E*)-β-ocimene synthase CsTPS6FN were the least abundant of all metabolites and transcripts measured, respectively. However, within transcript/metabolite pairs, only the correlation between (-)-limonene and *CsTPS1FN* transcript level was significant (Fig 6A). Correlation between metabolite level and transcript abundance was not significant for any of the other metabolite/transcript pairs.

In addition, we measured the transcript abundance of the multiproduct monoterpene synthase *CsTPS5FN*, to assess if its expression may contribute to terpene profiles in the resin. *CsTPS5FN* transcripts were highly abundant in some individuals, comparable to the highest transcript levels of any other *CsTPS* tested (Fig 6B). Transcript levels of this gene did not explain any of the lack of correlations between the five terpene-metabolite pairs tested above. Additionally, plant X, which had no detectable monoterpenes, had moderate levels of *CsTPS5FN* transcript. It appears that *CsTPS5FN*, while highly expressed, does not contribute to terpene accumulation in ‘Finola’.

## Discussion

The resin of *C*. *sativa* is rich in mono- and sesquiterpenes, which are of interest for their putative contributions to cannabis pharmacology [6]. Most studies of terpenes in cannabis have focused on phytochemical composition for forensics and breeding, while less research has gone into the molecular biology of terpene formation in cannabis. Knowledge of the genomics and gene functions of terpene biosynthesis may facilitate genetic improvement of cannabis for desirable terpene profiles. Using the hemp strain ‘Finola’ and its genome and transcriptome resources [24], we identified early isoprenoid pathway genes as well as specific *CsTPS* genes and their enzymes involved in the biosynthesis of nearly all of the different monoterpenes identified in extracts of the cannabis inflorescences, which are densely covered with terpene and cannabinoid accumulating glandular trichomes (Fig 1). One exception is terpinolene, for which a *CsTPS* has not yet been identified. The terpene profiles of cannabis can be explained by activities of both single-product and multi-product *CsTPS*. Individual ‘Finola’ plants showed substantial variation in their profiles of mono- and sesquiterpenes. ‘Finola’ has few monoterpene alcohols or ethers, such as linalool or geraniol, which are common in some cannabis strains.

**Fig 7:**
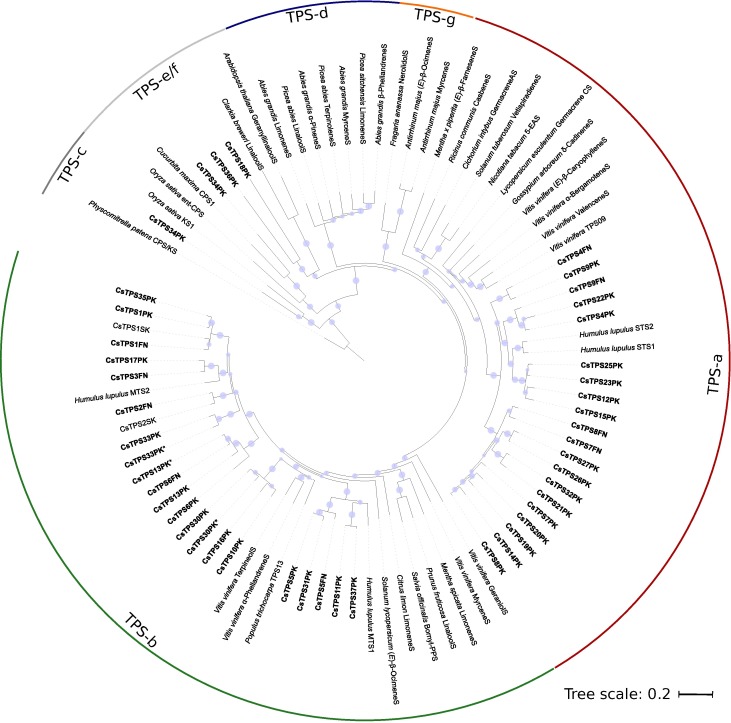
Maximum likelihood phylogeny for 33 TPS translated from gene models identified in the *Cannabis sativa* Purple Kush genomic sequences. 41 published TPS sequences from other organisms were included for comparison. Names of cannabis genes identified in this study are in bold. Gene names from Purple Kush followed by an asterisk (*) represent biochemically characterized enzymes from Purple Kush transcriptome data. Their nearest homologue in the genome was assigned the same gene ID when the sequences had >95% amino acid identity. Branches with greater than 80% boostrap support are identified with a grey circle.

It is reasonable to expect that there are additional *CsTPS* not described in this work, such as a *CsTPS* that encodes a terpinolene synthase. Characterization of further TPS may also clarify the poor correlation between TPS and the abundance of their products shown in Fig 6A. A search of a new assembly of the Purple Kush genome, to which we recently had pre-publication access (Dr. Jonathan Page, personal communication), identified a total of 33 complete *CsTPSPK* gene models and additional partial sequences (Fig 7). Purple Kush is a marijuana strain which requires special research licensing to grow. Thus, characterization of this more comprehensive set of *CsTPSPK* will have to be completed in future work as it requires synthesized genes.

Fig 7 indicates a set of putatively orthologous *CsTPSFN* and *CsTPSPK* genes, which may contribute to overlapping terpene profiles in hemp and marijuana varieties. However, some orthologous genes may have evolved different functions in different strains, and non-orthologous *CsTPS* may contribute to some of the same terpene products in different cannabis strains. For example, α-pinene is a major component of strains reported as Purple Kush [37], but no obvious orthologue of the α-pinene synthase CsTPS2 as identified in the ‘Finola’ and ‘Skunk’ strains was found in the Purple Kush genome (Fig 7). Another example is the set of apparently non-orthologous single-product myrcene synthases, CsTPS3FN and CsTPS30PK identified in ‘Finola’ and Purple Kush, which only share 52.5% amino acid identity but produce the same monoterpene. Also, not all *CsTPS* are expected to contribute to terpene accumulation in the resin of cannabis inflorescences and some may function in a different context of the plant biology. For example, *CsTPS5FN* is expressed in inflorescences and the recombinant enzyme produces a mixture of monoterpenes, but does not contribute substantially to the terpene profile of the resin. This gene appears most closely related to *MTS1* from hops (Fig 3) where the encoded protein was inactive *in vitro* [38].

Cannabis inflorescences are densely covered with glandular trichomes, which are specialized to produce and accumulate terpenes [11]. Transcripts of several *CsTPS* genes (Fig 6A) are abundant in trichomes isolated from mid-stage ‘Finola’ inflorescences. Transcripts associated with early isoprenoid biosynthesis and especially the MEP pathway, which feeds into both monoterpene and cannabionoid biosynthesis, were also abundant in trichomes (Fig 2B). Sesquiterpenes have been reported to be most abundant in early floral stages [39], and thus MEV pathway transcripts may be more abundant at earlier stages of flower development. Different DXS and HMGR genes were differentially expressed in roots relative to other parts of the plant. Terpenes in the roots, if present in cannabis, may contribute to defense as reported in other plant species [10]. In plants, *DXS* genes generally fall into two clades, of which DXS I members are generally involved in primary metabolism, and DXS II members are often induced in defense responses [40, 41, 42]. Abundance of cannabis *DXS2* transcripts, which clusters with the DXS II subfamily (S1 Fig), suggests defense related terpenoids in cannabis roots and warrants future work on the cannabis root metabolome. We also observed high *FPPS* transcript abundance in stamenate flowers and roots, resembling a previous finding that Arabidopsis FPS1 was primarily expressed in flowers and roots compared to AtFPS2 [43].

Domestication and selective breeding can result in changes in terpene profiles and abundance. For example, domestication can lead to a decrease in the quantity or variability of terpenes [44, 45, 46]. Cannabis, especially marijuana, has been domesticated for thousands of years for increased resin volume and potency [2, 47] and as a result profiles and ecological roles of terpenes in ancestral (i.e., undomesticated) cannabis are unknown. The present study highlights the large number of CsTPS genes and the diverse products of the encoded TPS enzyme activities, which contribute to the complex terpene profiles of cannabis. The knowledge of multigene nature of the CsTPS family and the often multiple products of the encoded enzymes will be critical when selecting or breeding, or improving plants by genome editing, for particular terpene profiles for standardized cannabis varieties. While cannabinoid-free individuals have occasionally been reported [48], we are not aware of any reports in the literature of terpene-free cannabis. In this study, we observed a single monoterpene-free individual, which however still contained cannabinoids and sesquiterpenes. This observation implies that biosynthesis of the different classes of terpenoid metabolites are independently regulated. The fact that terpenes have persisted throughout domestication as a substantial and diverse component of cannabis resin highlights their significance for human preferences.

## GenBank accessions

GenBank accession numbers for the terpene synthases described in this paper are CsTPS1FN: KY014557, CsTPS2FN: KY014565, CsTPS3FN: KY014561, CsTPS4FN: KY014564, CsTPS5FN: KY014560, CsTPS6FN: KY014563, CsTPS7FN: KY014554, CsTPS8FN: KY014556, CsTPS9FN: KY014555, CsTPS11FN: KY014562, CsTPS12PK: KY014559, CsTPS13PK: KY014558. Accession numbers for genes in the MEP pathway are CsDXS1: KY014576, CsDXS2: KY014577, CsDXR: KY014568, CsMCT: KY014578, CsCMK: KY014575, CsHDS: KY014570, CsHDR: KY014579. Accession numbers for genes in the MEV pathway are CsHMGS: KY014582, CsHMGR1: KY014572, CsHMGR2: KY014553, CsMK: KY014574, CsPMK: KY014581, CsMPDC: KY014566, CsIDI: KY014569. Prenyltransferase accession numbers are CsGPPS.ssu1: KY014567, CsGPPS.ssu2: KY014583, CsFPPS1: KY014571, CsFPPS2: KY014580. Accession numbers for genomic regions containing putative terpene synthases from Purple Kush are CsTPS1PK: KY624372, CsTPS4PK: KY624361, CsTPS5PK: KY624374, CsTPS6PK: KY624363, CsTPS7PK: KY624368, CsTPS8PK: KY624352, CsTPS9PK: KY624366, CsTPS10PK: KY624347, CsTPS11PK: KY624348, CsTPS12PK: KY624349, CsTPS13PK: KY624350, CsTPS14PK: KY624351, CsTPS15PK: KY624353, CsTPS16PK: KY624354, CsTPS17PK: KY624355, CsTPS18PK: KY624356, CsTPS19PK: KY624357, CsTPS20PK: KY624358, CsTPS21PK: KY624360, CsTPS22PK: KY624360, CsTPS23PK: KY624362, CsTPS24PK and CsTPS25PK: KY624364, CsTPS26PK and CsTPS27PK: KY624365, CsTPS30PK: KY624367, CsTPS31PK: KY624369, CsTPS32PK: KY624370, CsTPS33PK: KY624371, CsTPS34PK: KY624373, CsTPS35PK: KY624375.

## Supporting information

S1 Table Primers used to clone *TPS* genes. (XLSX)

S2 Table qPCR primers. (XLSX)

S3 Table Accession numbers of TPS sequences used in tblastn and to construct phylogeny. (XLSX)

S1 Fig DXS Phylogeny. Maximum likelihood phylogeny of DXS enzymes. *Cannabis sativa* genes are in bold. DXS of other species included are from: At: *Arabidopsis thaliana*; Pt: *Populus trichocarpa*; Os: *Oryza sativa*; Cr: *Chlamydomonas reinhardtii*; Mt: *Medicago truncatula*; Pa: *Picea abies*.
(PNG)

S2 Fig Representative GC-MS traces of myrcene synthase products. (PNG)

S3 Fig Mass spectra of TPS products. Labels “Peak a” through “Peak u” correspond to peaks labeled in Figs 4 and 5.
(PNG)

S4 Fig Products of CsTPS5FN expressed in *E*. *coli and Nicotiana benthamiana*. Black trace represents products of recombinant enzyme expressed in *E*. *coli*, green trace represents products of recombinant enzyme expressed in *N*. *benthamiana*. Leaf images (right) show GFP positive expression control.
(PDF)

S5 Fig Products of CsTPS with alternative substrates. Members of TPS-a with GPP as substrate are on the left-hand side. Members of TPS-b with FPP as substrate are on the right.
(PDF)

S6 Fig Hot vs. cold injection of CsTPS8FN products. Top panel represents total ion chromatogram (TIC) with the injection port at 250°C on a DB-Wax column. The bottom panel represents TIC with the injection port at 40°C, using the same program and the same column.
(PDF)

S7 Fig Isolated glandular trichome heads. (PDF)

S8 Fig Terpene chemotypes of ‘Finola’ flowers. Abundance of five metabolites or metabolite pairs is measured relative to floral weight and an internal standard. Error bars indicate the standard deviation of five metabolite samples taken from each individual.
(PDF)

S9 Fig Amino acid sequence alignment of functionally characterized CsTPS enzymes. (PDF)
