# Supplementary figures and images for: Terpene synthases from Cannabis sativa
Source: PLoS One. 2017 Mar 29;12(3):e0173911. doi: 10.1371/journal.pone.0173911 (PMC5371325; doi:10.1371/journal.pone.0173911)

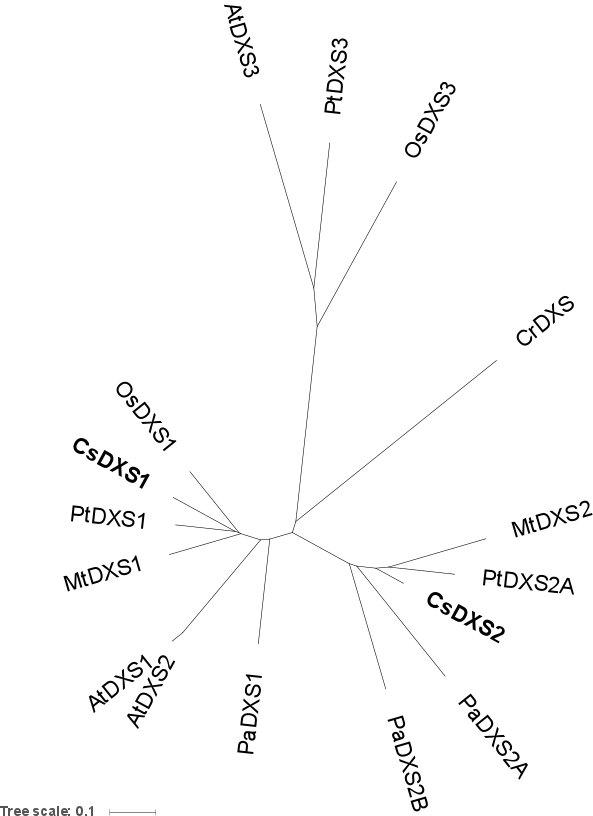

Supplement: S1 Fig — Maximum likelihood phylogeny of DXS enzymes. Cannabis sativa genes are in bold. DXS of other species included are from: At: Arabidopsis thaliana; Pt: Populus trichocarpa; Os: Oryza sativa; Cr: Chlamydomonas reinhardtii; Mt: Medicago truncatula; Pa: Picea abies. (PNG) [file pone.0173911.s004.png]

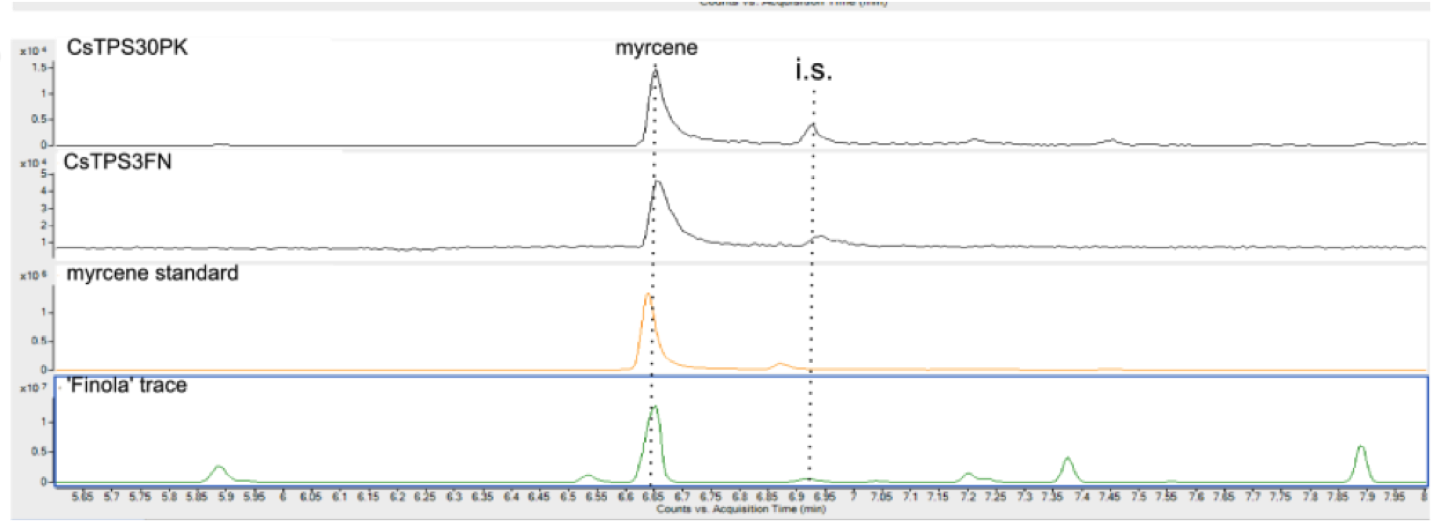

Supplement: S2 Fig — (PNG) [file pone.0173911.s005.png]

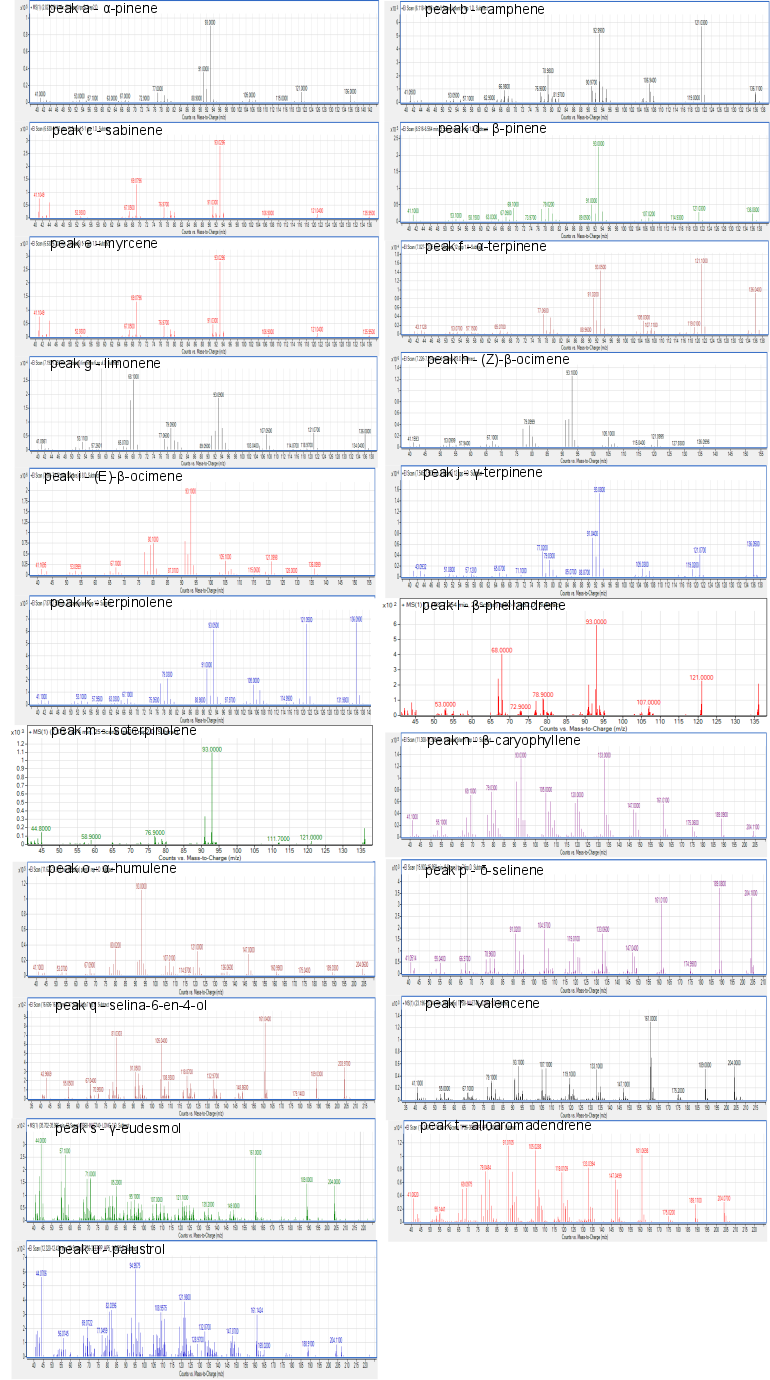

Supplement: S3 Fig — Labels “Peak a” through “Peak u” correspond to peaks labeled in Figs 4 and 5. (PNG) [file pone.0173911.s006.png]

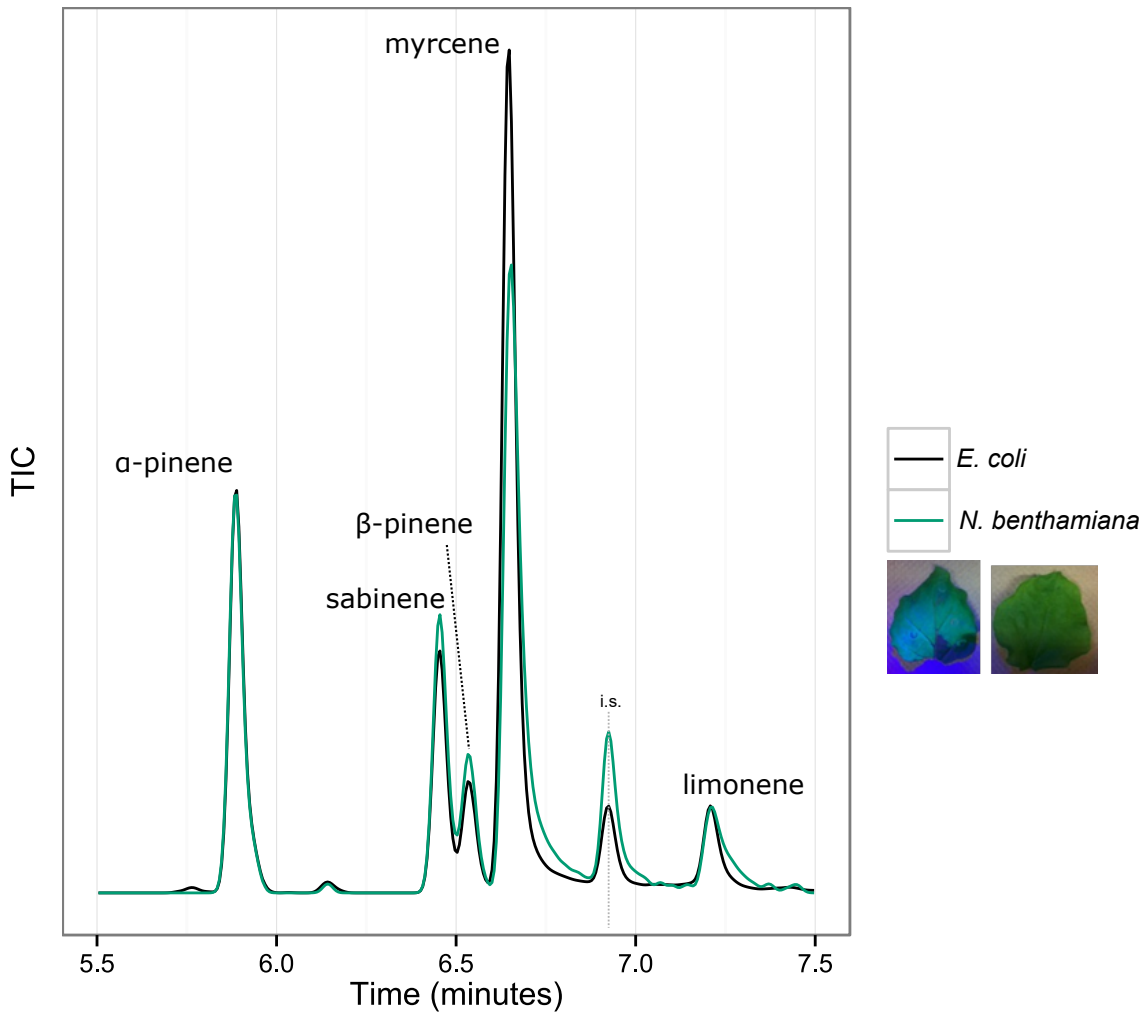

Supplement: S4 Fig — Black trace represents products of recombinant enzyme expressed in E. coli, green trace represents products of recombinant enzyme expressed in N. benthamiana. Leaf images (right) show GFP positive expression control. (PDF) [file pone.0173911.s007.pdf]

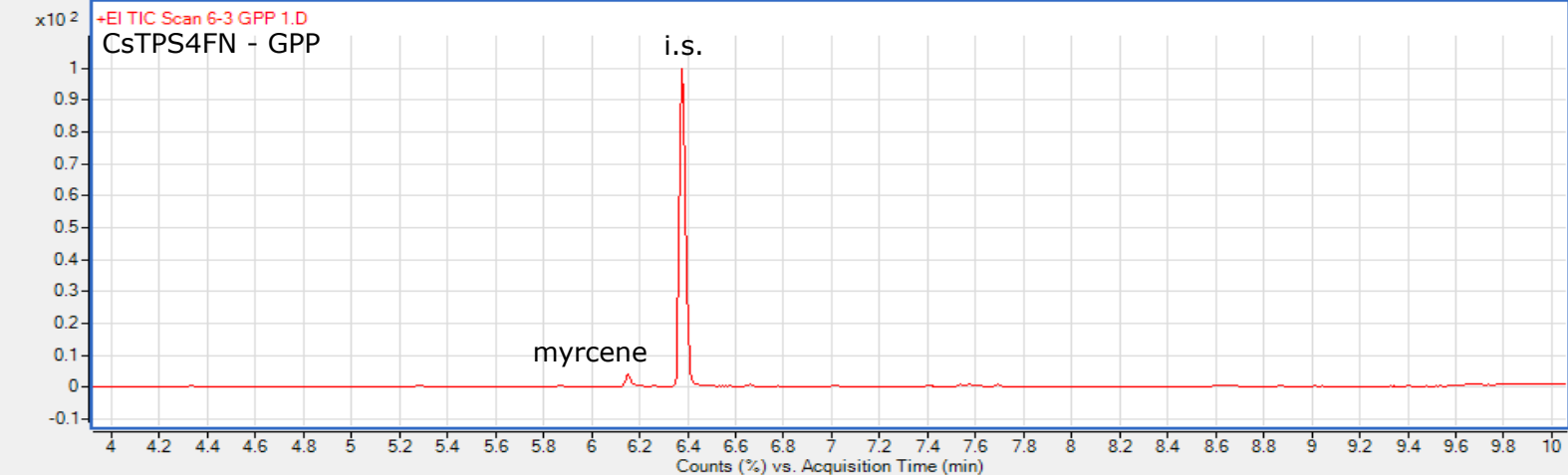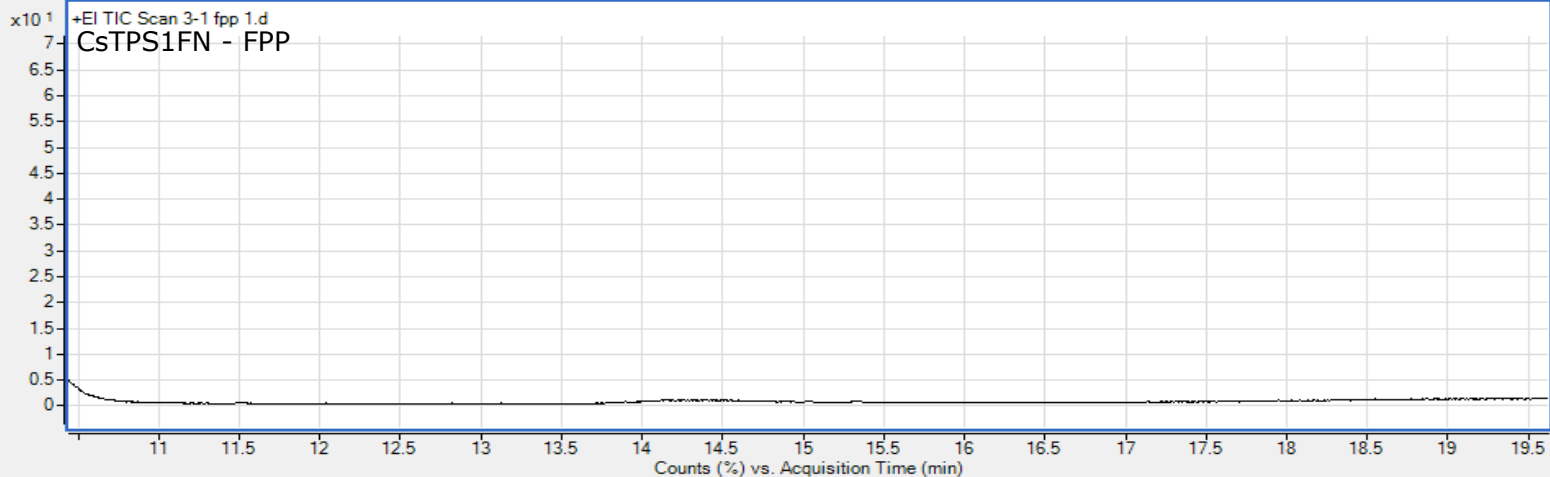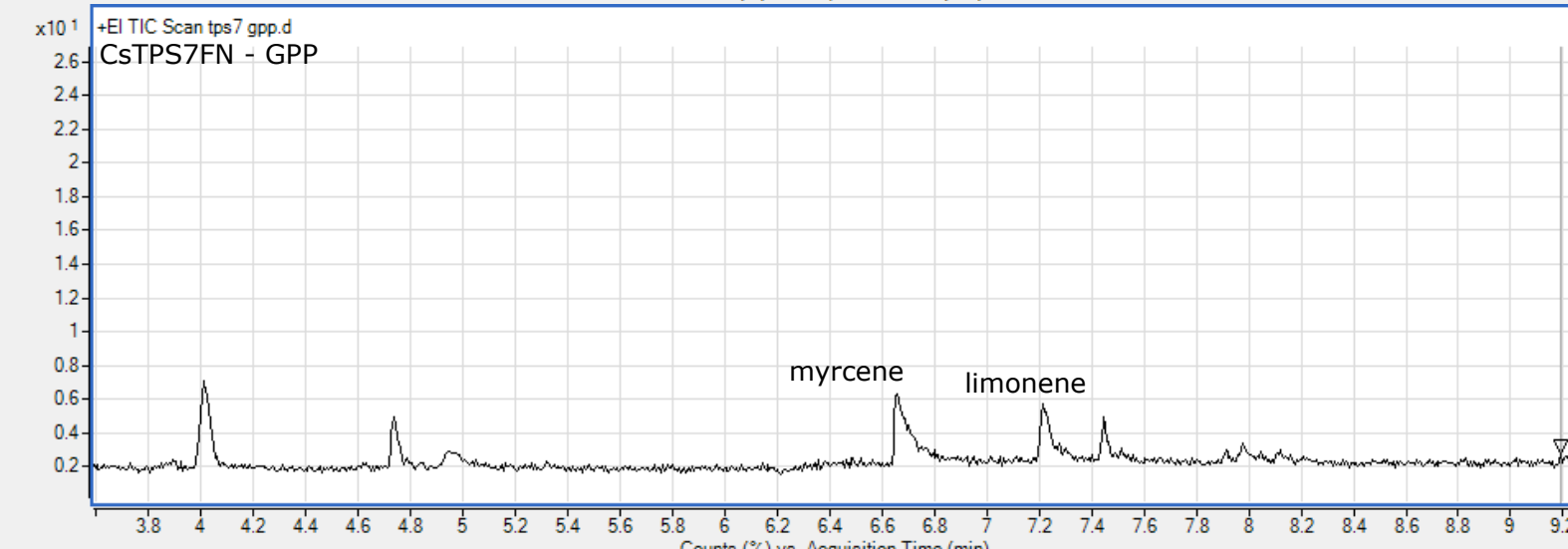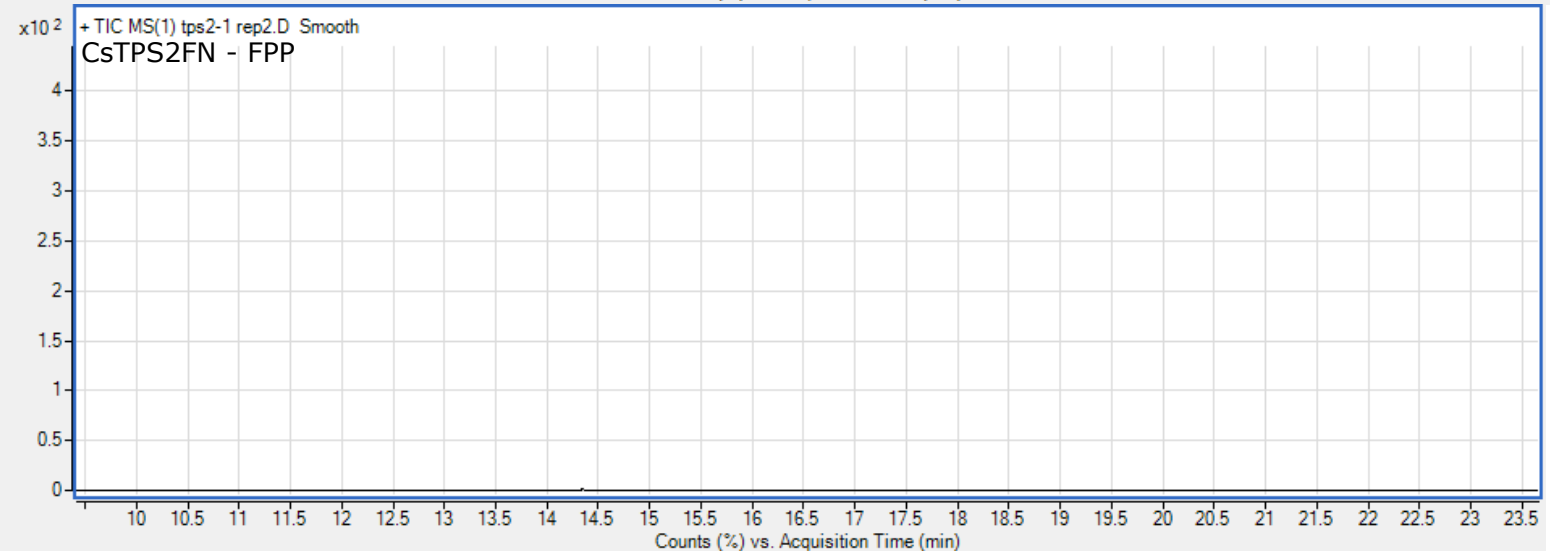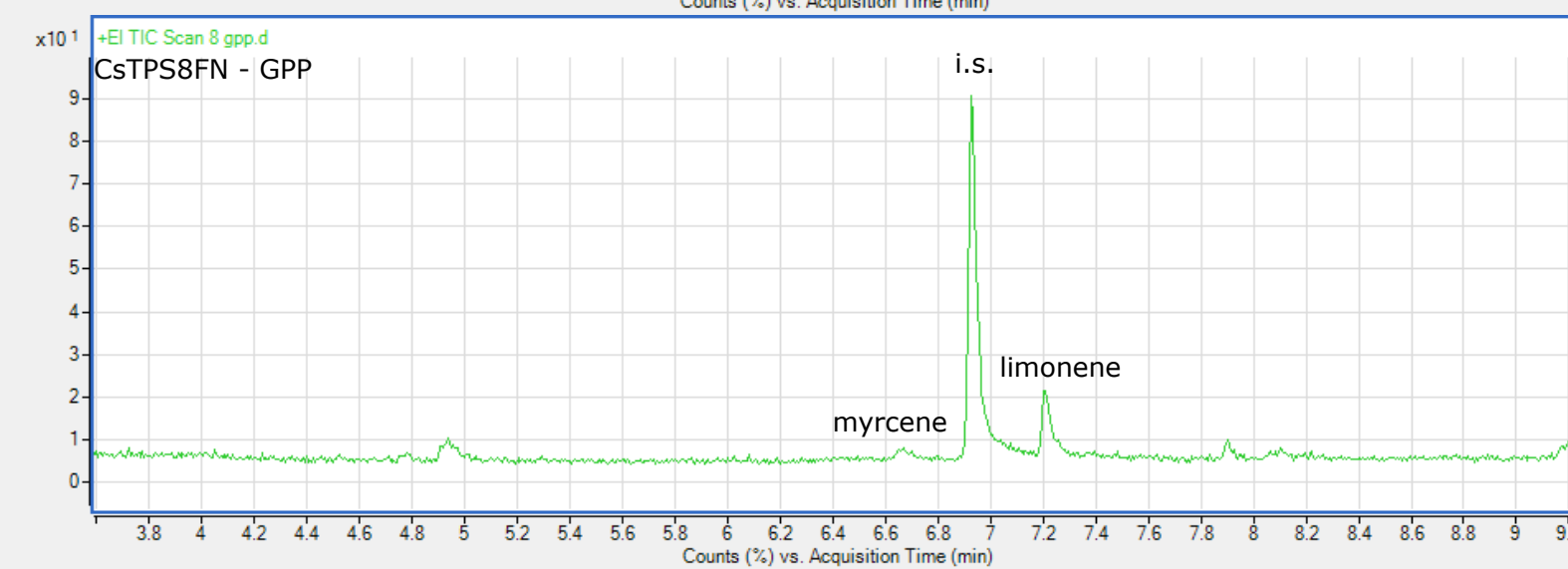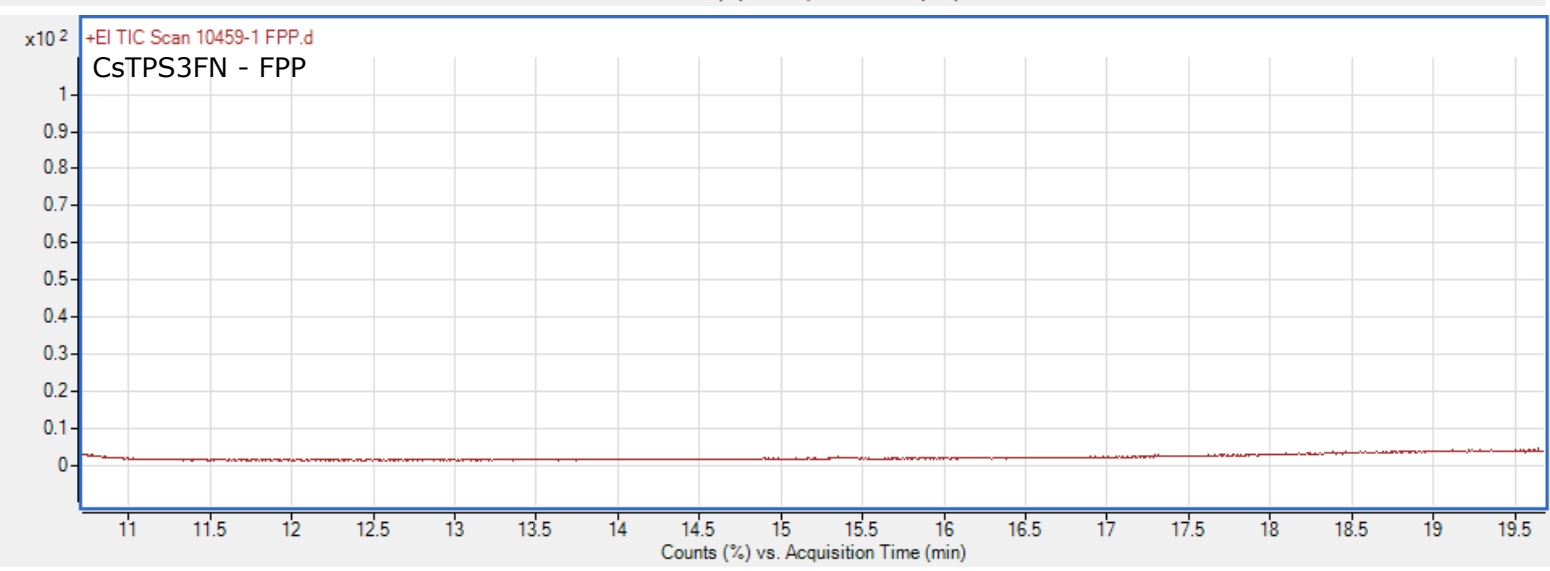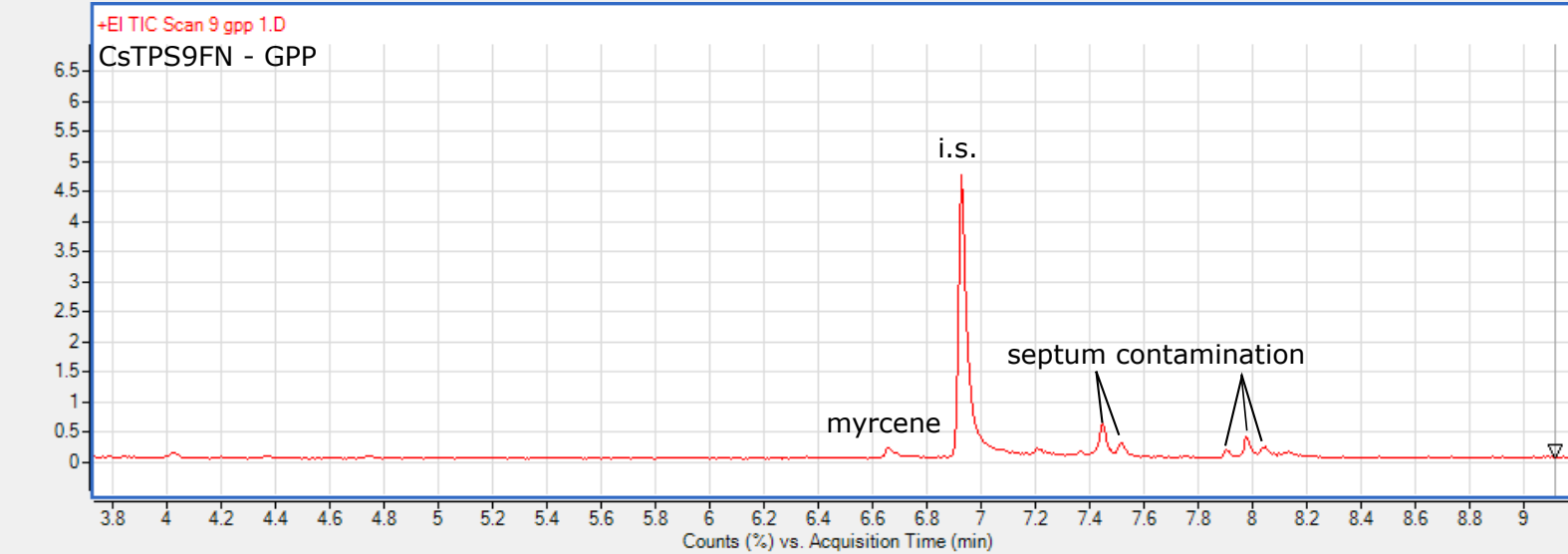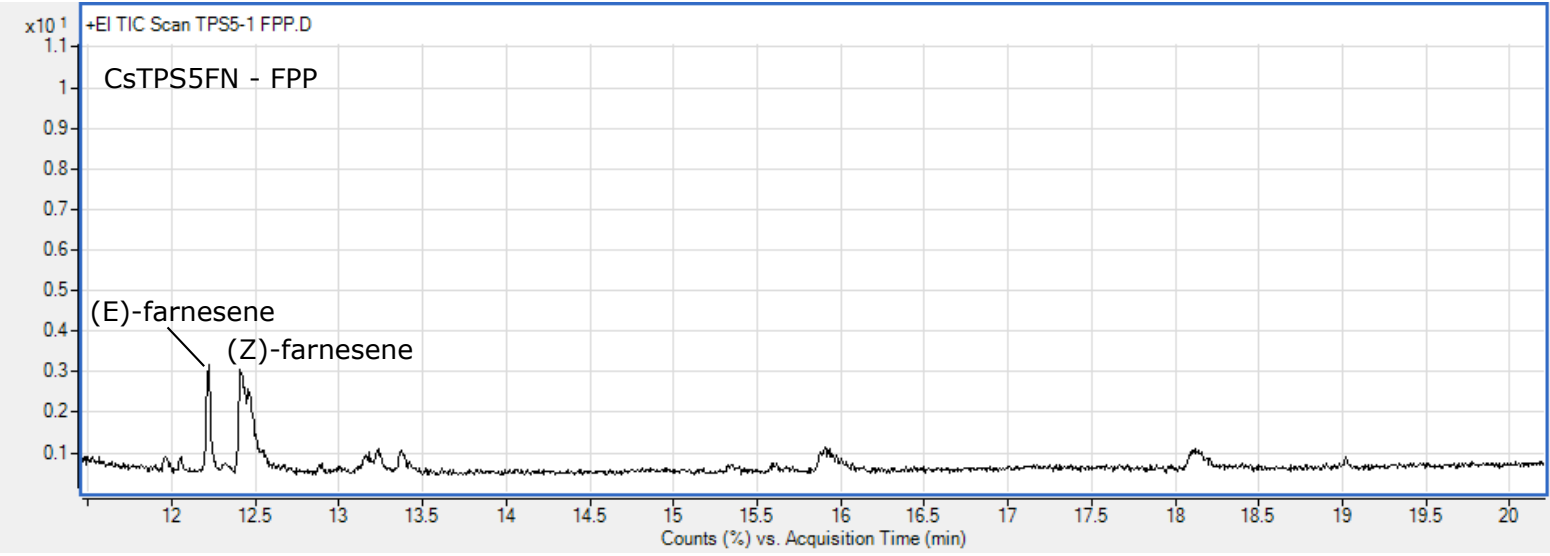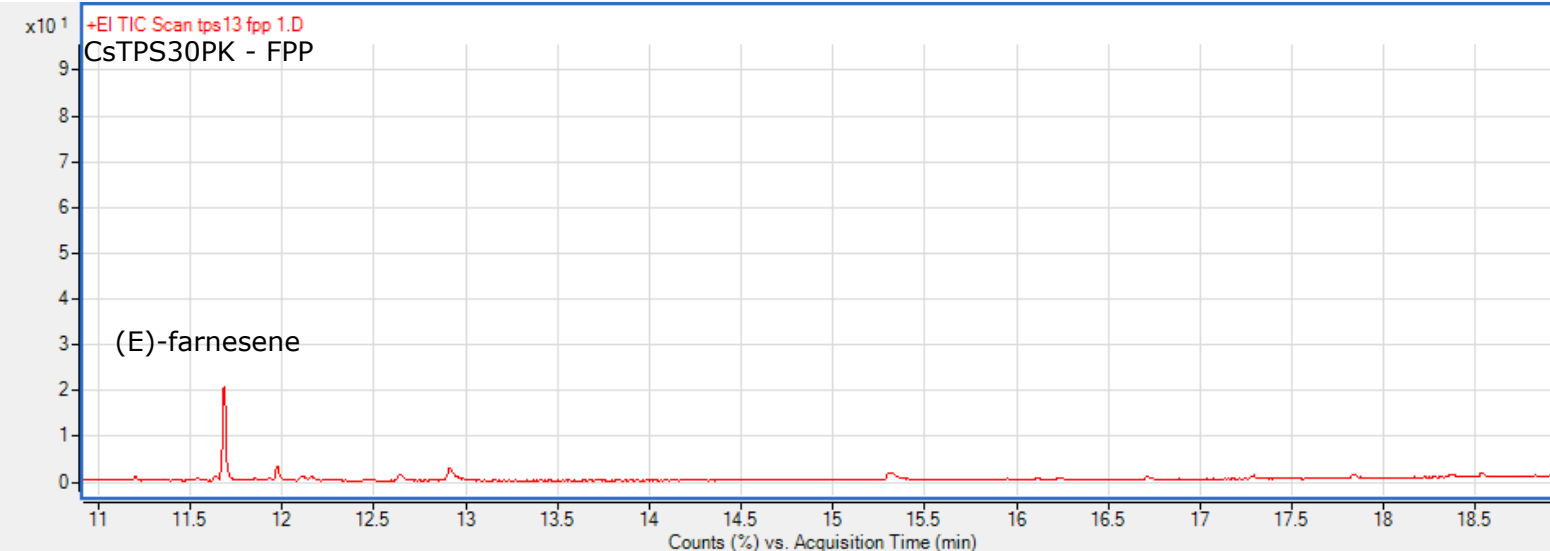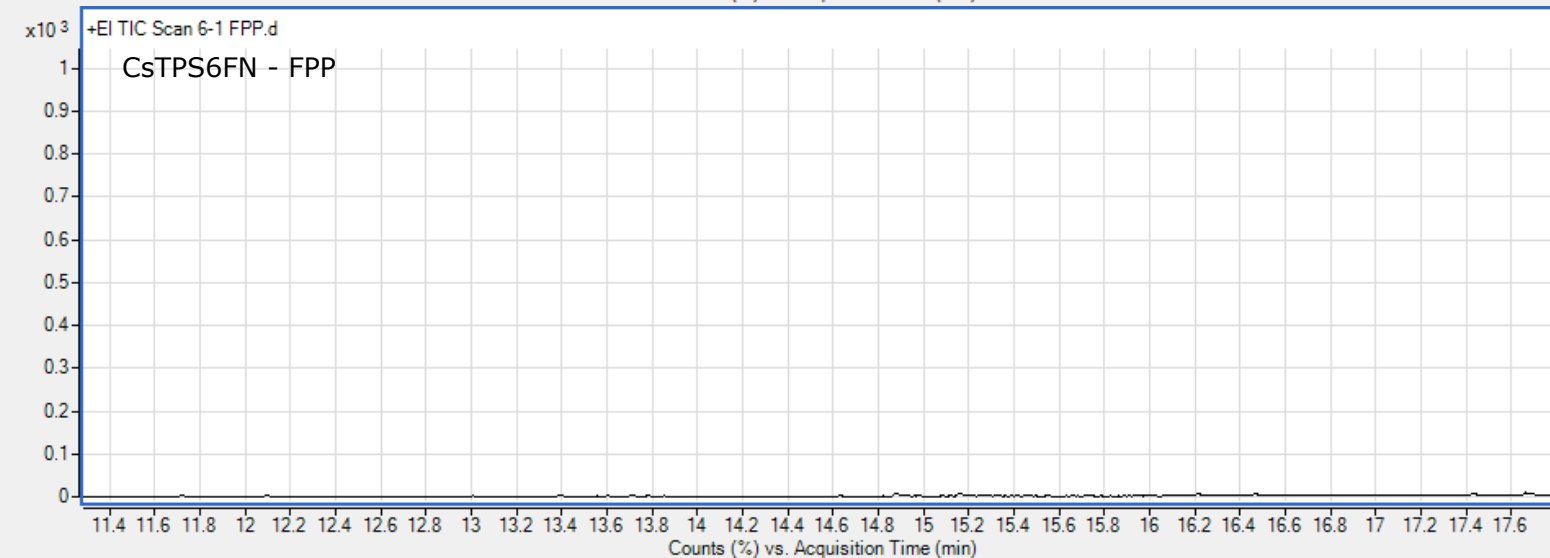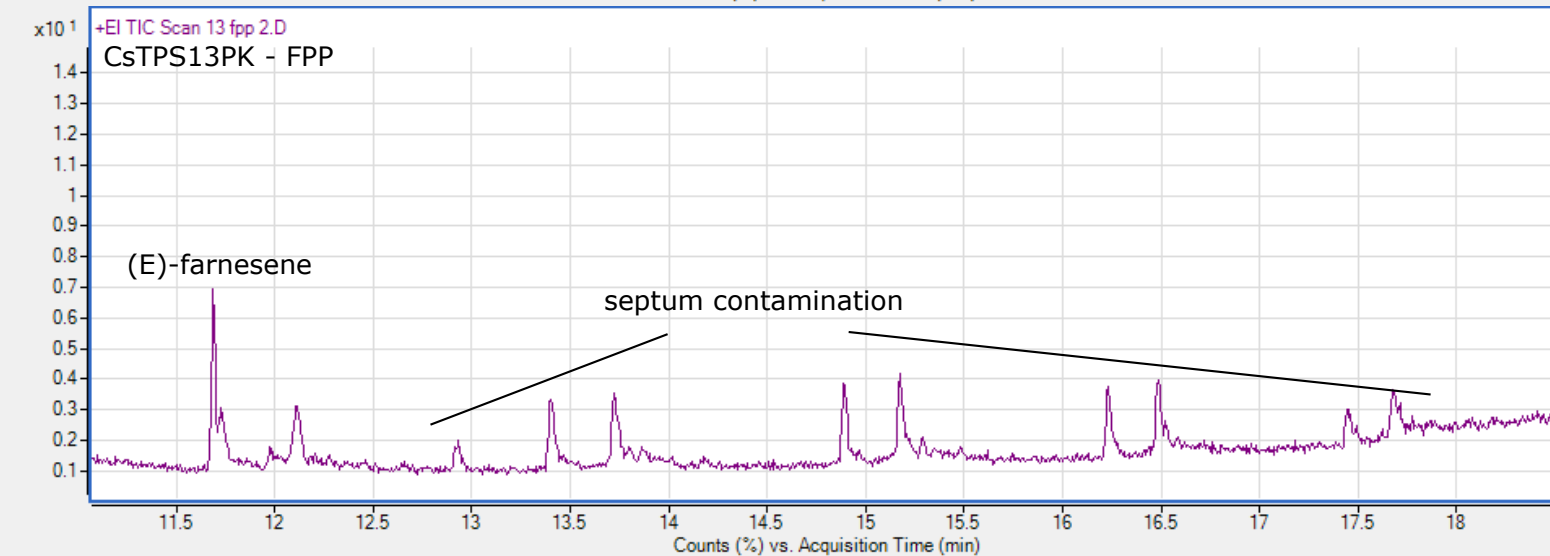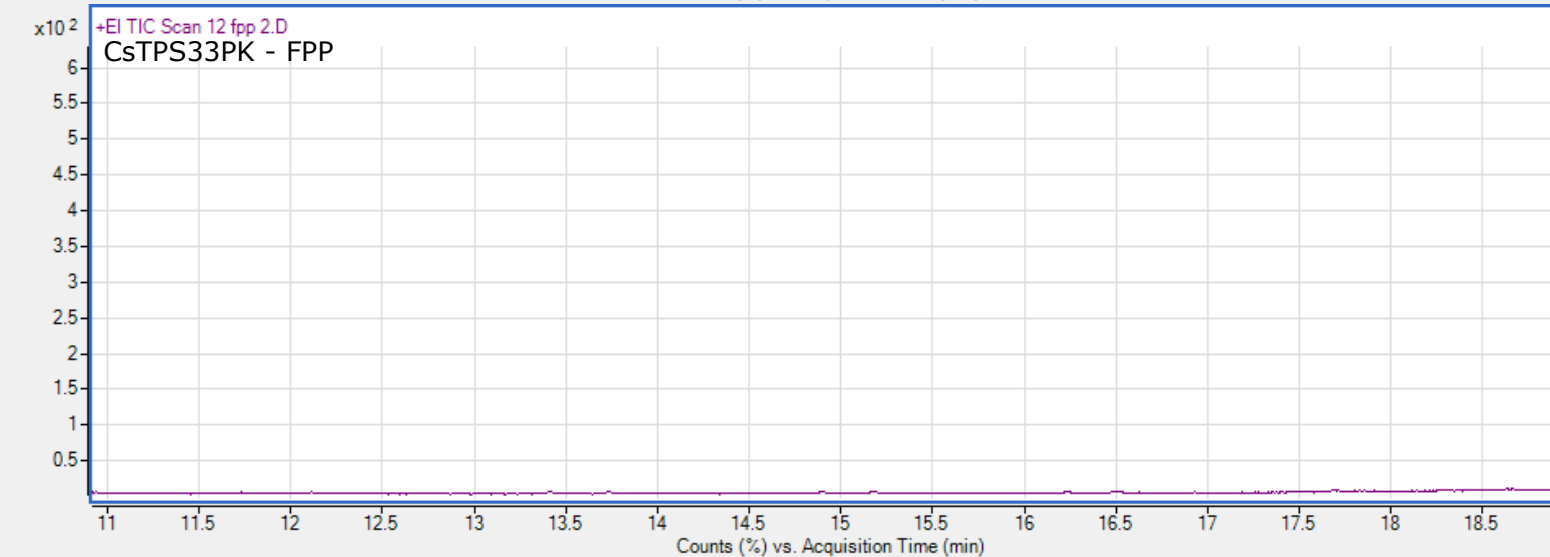

Supplement: S5 Fig — Members of TPS-a with GPP as substrate are on the left-hand side. Members of TPS-b with FPP as substrate are on the right. (PDF) [file pone.0173911.s008.pdf]

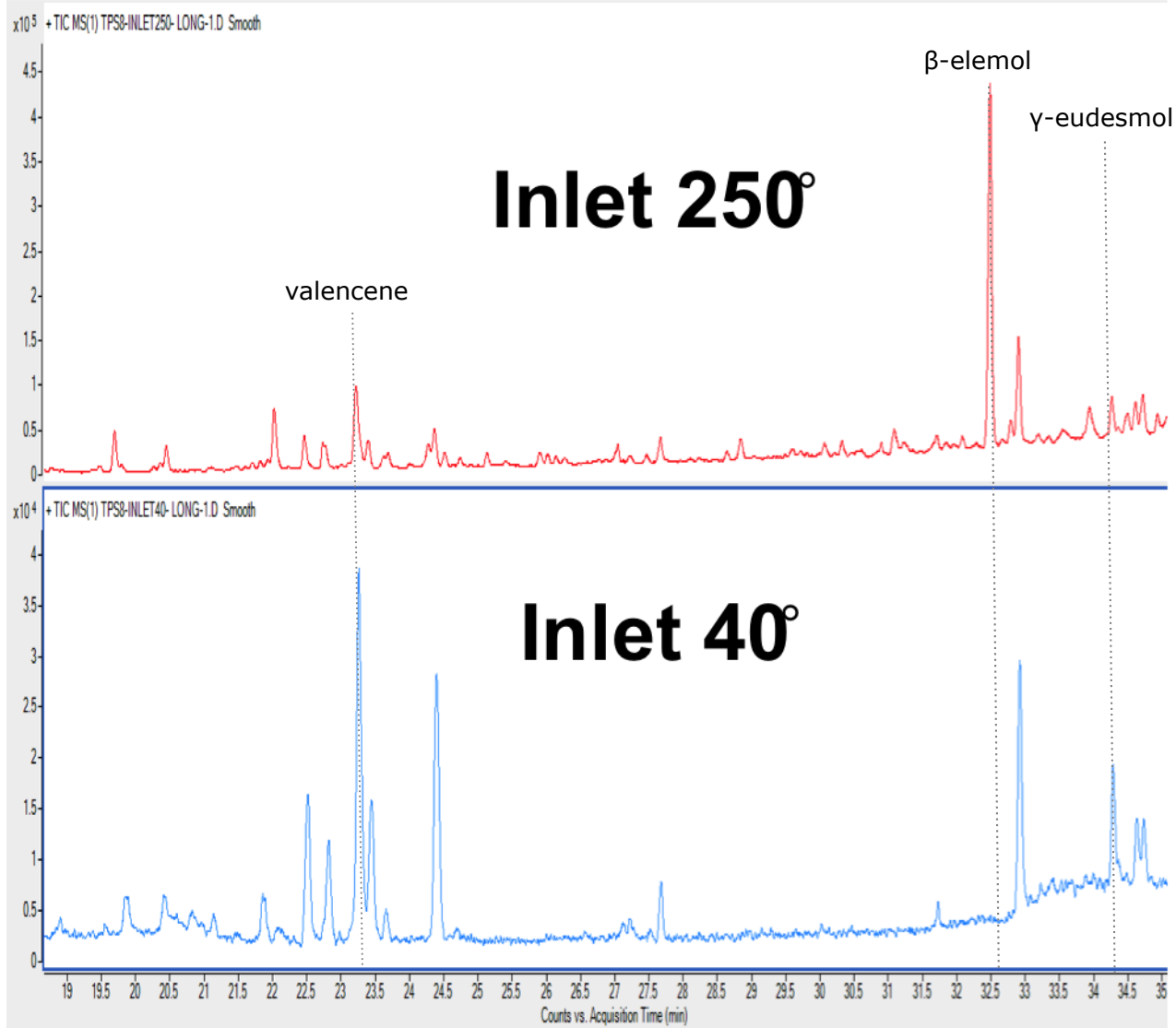

Supplement: S6 Fig — Top panel represents total ion chromatogram (TIC) with the injection port at 250°C on a DB-Wax column. The bottom panel represents TIC with the injection port at 40°C, using the same program and the same column. (PDF) [file pone.0173911.s009.pdf]

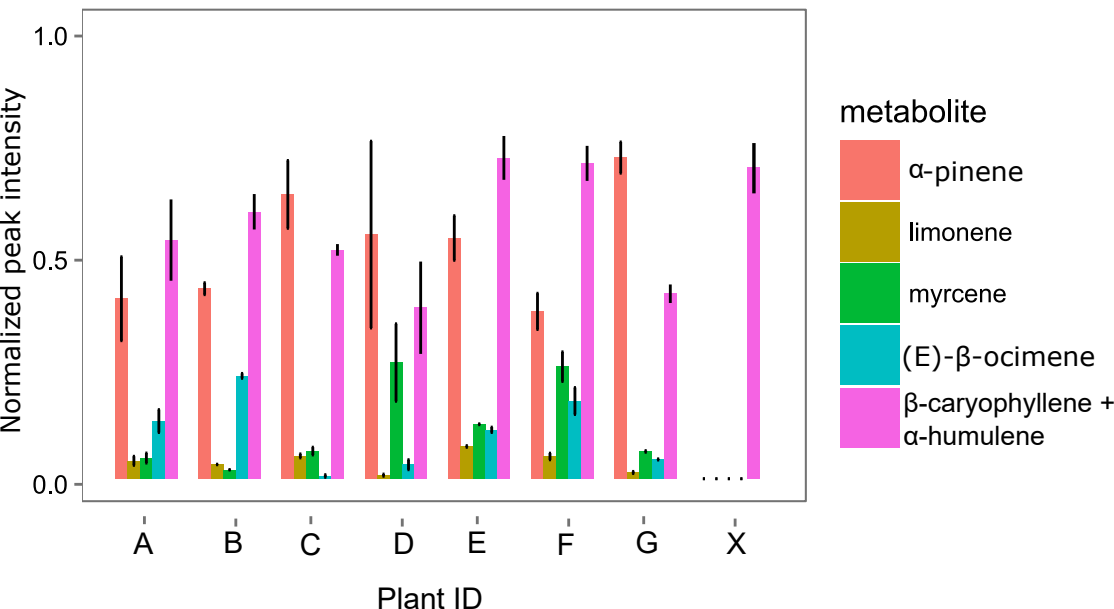

Supplement: S8 Fig — Abundance of five metabolites or metabolite pairs is measured relative to floral weight and an internal standard. Error bars indicate the standard deviation of five metabolite samples taken from each individual. (PDF) [file pone.0173911.s011.pdf]
