# Supplementary material for: Terpene synthases from Cannabis sativa
Source: PLoS One. 2017 Mar 29;12(3):e0173911. doi: 10.1371/journal.pone.0173911 (PMC5371325; doi:10.1371/journal.pone.0173911)
Supplement: S7 Fig — (PDF) [file pone.0173911.s010.pdf]

```
                            20                  40
CsTPS3FN   M--AVHQFSPSIVSSLPTISTYNNNHFCRFFTPKTSI-SPISKTKSK-------- 44
CsTPS1FN   M--AFHQFAPS--SSLPIWSRVNNP-----FTPKTSIITSISKPKSKPKSNLKSR 46
CsTPS2FN   M------------------------------------------------------ 1
CsTPS33PK  MRLGVHQFSP--------LSLILN-------TTKLARASTLS------------- 27
CsTPS13PK  MLY-----------------------------------------RPKIYTNYNII 14
CsTPS6FN   MLY-----------------------------------------RPKIYTNYNII 14
CsTPS30PK  M------------------------------------AALVSTVSSIIRWNSNNN 19
CsTPS5FN   MSL---------------------------------------------------- 3
CsTPS8FN   MS----------------------------------------------------- 2
CsTPS7FN   MS----------------------------------------------------- 2
CsTPS9FN   MS----------------------------------------------------- 2
CsTPS4FN   MS----------------------------------------------------- 2

               60                  80                  100
CsTPS3FN   --------SSTCYPIQCTVVN--NSSPSSTIV---------------RRSANYEP 74
CsTPS1FN   SKLRSRSRSSTCYPIQCIVID--N--PSSTII----------NNS-DRRSANYGP 86
CsTPS2FN   ----------------CSLAKSPS-SDTSTIV---------------RRSANYDP 24
CsTPS33PK  ---------SACYPIQCTMVNSTNITNNTTIF----------DNHIHRRSANYEP 63
CsTPS13PK  NGGTKSRLSSACYPIQCAVVNSSN-----AII----------D----RRSANFEP 50
CsTPS6FN   NGGTKSRLSSACYPIQCAVVNSSN-----AII----------D----RRSANFEP 50
CsTPS30PK  NNNNFTRSVKSC--LSSNYHNNNNIIHNKTVLMSTNNNNNNNQKNSSRRSANYQP 72
CsTPS5FN   ----------------SGLISTTTFKEQPAIV---------------RRSGNYKP 27
CsTPS8FN   ---------------NIQVLASSQLCDKI--IA--------------RPTTKFHP 26
CsTPS7FN   ---------------S-QVLASSQLSDKI--IA--------------RPTTNFHP 25
CsTPS9FN   ----------------YQVLASSQ-NDKVSKIV--------------RPTTTYQP 26
CsTPS4FN   ---------------T-QILATSFKNDNIHKIV--------------RPTTNYHP 27

                    120                 140                 160
CsTPS3FN   PIWSFDYIQ---SLST-QYKGESYTRQLNKLKKEVKRMLL-RMEINS-LALLELI 123
CsTPS1FN   PIWSFDFVQ---SLPI-QYKGESYTSRLNKLEKDVKRMLI-GVE-NS-LAQLELI 134
CsTPS2FN   PIWSFDFIQ---SLPC-KYKGEPYTSRSNKLKEEVKKMLV-GME-NS-LVQLELI 72
CsTPS33PK  PIWSFDYIQ---SLSSSQYKGETCTSRLNELEANVKEILLVEMKSNS-LAQLEFI 114
CsTPS13PK  SIWSFDYIQ---SL-TSQYKGEPYTSRVKKLERDVKKILV-EME-NS-LAQLELI 98
CsTPS6FN   SIWSFDYIQ---SL-TSQYKGEPYTSRVKKLERDVKKILV-EME-NS-LAQLELI 98
CsTPS30PK  PLWQFDYVQ---SL-SSPFKDEAYVKRVEKLKEEVRVMVKRAREEEKPLSQLELI 123
CsTPS5FN   PLWDAHFIQ---SLQVI-YTEESYGKRINELKEDVRRIL--EKEAENPLVKLEQI 76
CsTPS8FN   SIWGDRFLHYNISEQDLVCKQE----RVEELIQVVKKEILSSN-----HDQLKLI 72
CsTPS7FN   SIWGDRFLHYNVSEQDLVCKQE----RIEELIQVVKKEILSSN-----HDQLKLI 71
CsTPS9FN   SIWGERFLQYSISDQDFSYKKQ----RVDELKEVVRREVFLE-CYDNVSYVLKIV 76
CsTPS4FN   SIWGDRFLHYDIPKEELNYKQG----QVEELKEVVRKEIFGEFLCDDWNNRLKLI 78

                         180                 200                 220
CsTPS3FN   DTLQRLGISYHFKNEINTILKKKYNDNYINNNIITNPNYNN-LYATALEFRLLRQ 177
CsTPS1FN   DTIQRLGISYRFENEIISILKEKFTNN--NNN--PNPNYD--LYATALQFRLLRQ 183
CsTPS2FN   DTLQRLGISYHFENEIISILKKYFTNISTNKN----PKYD--LYATALEFRLLRE 121
CsTPS33PK  DALQRLGLSYRFETEINTILNEKYS-----GNIIDNPNYN--LYATALEFRLLRH 162
CsTPS13PK  DTLQRLGISYRFENEINSILNKKYLN-------INNPNYSDLLYAIALEFRLLRQ 146
CsTPS6FN   DTLQRLGISYRFENEINSILNKKYVN-------INNPNYN--LYAIALEFRLLRQ 144
CsTPS30PK  DVLQRLGISYHFEDEINDILKHIYNNNNVY-------NTNNNVYANSLEFRLLRQ 171
CsTPS5FN   NDLSRLGISYHFEDQIKAILNLTYNNNNALWK-------KDNLYATALHFKLLRQ 124
CsTPS8FN   DNLQRLGLSYHFESEIEKLLE----QLSI--GTHHQNN---NLHDVSLRFRLLRQ 118
CsTPS7FN   DNLQRLGLSHHFESEIEKLLE----QLSI--GTHHQNH--QDLHDASLWFRLLRQ 118
CsTPS9FN   DDVQRLGLSYHFENEIEKALQHIYD------NTIHQNHKDEDLHDTSTRFRLLRQ 125
CsTPS4FN   DVVERLGLSYHFESEIQNELQHIYNKISI--NDSNFKH--ETLHDASIRFRLLRQ 129

                              240                 260
CsTPS3FN   HGY-TVPQEIFNAFKD-KRGKFKTSLS-DDIMGVLCLYEASFYAMKHEN-ILEEA 228
CsTPS1FN   YGF-EVPQEIFNNFKNHKTGEFKANIS-NDIMGALGLYEASFYGKKGES-ILDEA 235
CsTPS2FN   YGY-AVPQEIFNDFKDE-TGKFKASIKNDDIKGVLALYEASFYVKNGEN-ILEEA 173
CsTPS33PK  HGY-DVSQEIFNVFKDEITSKFKARTSGEDIIGVLALYEASFYGKKSES-ILEEA 215
CsTPS13PK  HGY-AVPQEIFNQLKNEIEN-IKKNINGNDIMGILALYEASFYEKKCES-ILKEA 198
CsTPS6FN   HGY-AVPQEIFNQLKNEIEN-IKKNINGNDIMGILALYEASFYEKKCES-ILKEA 196
CsTPS30PK  HGY-PVSQEIFSTCKDERGNFM---VCTNDIKGMLSLYEASFYLVENEDGILEET 222
CsTPS5FN   YGFNPVSSEIFNAFKDEKK-EFKESLS-KDVKGMVCLYEASFYSFRGEP-ILDEA 176
CsTPS8FN   SGFK-VSSSIFEKFKDE-KGNFKESLI-TDVSGLLSLYEASHLSYVGES-ILDEA 169
CsTPS7FN   HGLN-VSSSIFEKFKDD-EGNFKKSLI-TDVSGLLSLYEASHLSYVGES-ILDEA 169
CsTPS9FN   HGFM-VSSNIFKIFKDE-QGNFKECLI-TDILGLLSLYEASHLSYIGEN-ILNEA 176
CsTPS4FN   HGYR-VSLDIFDKFKDE-NGNFKECLA-SDTVGLLSLYEASHLSCVGEN-LLDEA 180

               280                 300                 320
CsTPS3FN   RIFSTKCLKKYMEKIENEEEKKILLLND-NNINSNLLLINHAFELPLHWRITRSE 282
CsTPS1FN   RIFTTKCLKKY---------KLMSSSNN-NNMTLISLLVNHALEMPLQWRITRSE 280
CsTPS2FN   RVFTTEYLKRYVMMIDQN-----MILND-----NMAILVRHALEMPLHWRTIRAE 218
CsTPS33PK  RVSSIECLENYVATMTMERNKPSLLVNDYDDDNNMLLLVNHALELPLYWRITRSE 270
CsTPS13PK  RIFTTECLKNYTIMIS--EQKKLMIDNDYDYD---IEVVNHALELPLHRRTTRTE 248
CsTPS6FN   RIFTTECLKNYTIMIS--EQKKLMIDNDYDYD---IEVVNHALELPLHRRTTRTE 246
CsTPS30PK  REKTKKYLEEYIIMIMEKQQSLLDQNNNNYDYDYDYELVSHALELPLHWRMLRLE 277
CsTPS5FN   RDFTTKHLKQYLMMTRQGQNVDHDDDND-----LMVKLVEHALELPVHWRMKRLE 226
CsTPS8FN   LAFTTTHLKAIVANSK-----DHPLSHQ----------ISKALETPLRKTIERLH 209
CsTPS7FN   LAFTTTHLKSIVANSK-----NHPLSHQ----------ISKALERPLRMTLERLH 209
CsTPS9FN   LAFTTTHLHQFVKNEK-----THPLSNE----------VLLALQRPIRKSLERLH 216
CsTPS4FN   LSFTTKHLTEFLENNKKEHPNDDPLSKE----------ISRALERPLRKTLVNLH 225
```

```
                                   340                       360                       380
CsTPS3FN   ARWFIDEIYEKKQD--MNSTLFEFAKLDFNIVQSTHQEDLQHLSRWWRDCKLGG- 334
CsTPS1FN   AKWFIEEIYERKQD--MNPTLLEFAKLDFNMVQSTYQEELKLLSRWWKDSKLGE- 332
CsTPS2FN   AKWFIEE-YEKTQD--KNGTLLEFAKLDFNMLQSIFQEDLKHVSRWWEHSKLGKN 270
CsTPS33PK  ARWFID-LYEKNHN--MNSTLLEFAKLDYNMVQSIYQEDLKHLSRWWSHTKLGE- 321
CsTPS13PK  AKWFIDA-YAKKQD--MNPMLLELAKLDFNIVQSTHHEDLKHIFRWWRHTKLGE- 299
CsTPS6FN   AKWFIDA-YAKKQD--MNPMLLELAKLDFNIVQSTHHEDLKHIFRWWRHTKLGE- 297
CsTPS30PK  SRWFID-VYEKRLD--MNPTLLTLAKLDFNIVQSIYQDDLKHVFSWWESTDMGK- 328
CsTPS5FN   ARWFIDMYAEMSHHHHMNSTFLQLAKLDFNVVQSTYQEDLKHVVRWWKTTSLGE- 280
CsTPS8FN   ARFYIS-IYEK--DASHNKLLLELAKLDFNVLQCFHKKELSEIMRWWKEHEFVK- 260
CsTPS7FN   ARFYIS-IYEK--DASHNKVLLELAKLDFNLLQCFHKKELSEIVRWWKEHEFAK- 260
CsTPS9FN   ARHYIS-SYEN--KISHNKTLLELAKLDFNLLQCLHRKELSQISRWWKEIDFVH- 267
CsTPS4FN   ARYFIS-IYEK--DASHNKVLLQLAKLDFNLLQSMHKKELSEISRWWKELDSAH- 276

                     400                       420                       440
CsTPS3FN   KLNFARDRLMEAFLWDVGLKFEGEFSYFRRTNARLFVLITIIDDIYDVYGTLEEL 389
CsTPS1FN   KLPFVRDRLVECFLWQVGVRFEPQFSYFRIMDTKLYVLLTIIDDMHDIYGTLEEL 387
CsTPS2FN   KMVYARDRLVEAFLWQVGIRFEPQFSHFRRISARIYALITIIDDIYDVYGTLEEL 325
CsTPS33PK  KMDFFRDRLMECFLWTVGIACEPEKSYYRRMSGRLYVLITTIDDIYDVYGTLEEL 376
CsTPS13PK  KLNFARDRLMECFLWKVGIRFEPKFSYFRITTAKLFELITVIDDIYDVYGTLDEL 354
CsTPS6FN   KLNFARDRLMECFLWNIGIRFESKFSYFRTKTAKLFELVTFIDDIYDVYGTLDEL 352
CsTPS30PK  KLEFARDRTMVNFLWTVGVAFEPHFKSFRRMITKVNALITVIDDIYDVYGTLDEL 383
CsTPS5FN   RLPFARDRIVEIFLWSVGLKFEPQFRYCRKMLTKIGQLVTTMDDIFDVYGTLDEL 335
CsTPS8FN   KFPFARDRMVELYFWILGVYYEPKYSRARKLLTKVIALTSITDDTYDAYGTIDEL 315
CsTPS7FN   KFPFARDRMVELYFWILGVYYEPKYSRARKLLTKVIALTSITDDIYDAYGTIDEL 315
CsTPS9FN   KLPFARDRIVELYLWLLGVFHEPELSLARIISTKVIALASVADDIYDAYGTFEEL 322
CsTPS4FN   NFPFARNRIVELYIWILGVYYEPQYSFARNILVKIIALSSIADDIYDSYGIFEEH 331

                          460                       480
CsTPS3FN   ELFTSAVERWDVKLINEL-PDYMKMPFFVLHNTINEMGFDVLVEQNF-VNIEYLK 442
CsTPS1FN   QLFTNALQRWDLKELDKL-PDYMKSAFYFTYNFTNELAFDVLQEHGF-VHIEYFK 440
CsTPS2FN   ELFTKAVERWDVKTVDEL-PDYMKLPFFTLFNTVNEMAYDVLEEHNF-VSVEYLK 378
CsTPS33PK  ELFTNAVERWDVKAMDDL-PEYMRMPFFLLHNTINEMAFDVLGHQNF-LNVKFLK 429
CsTPS13PK  ELFTKAVERWDVKMINEL-PEYMKMPYLVLHNTINDMVFEVLRDQEISINIQYLK 408
CsTPS6FN   ELFTKAVERWDVKMINEL-PEYMKMPYLVLHNTINDMVFEVLRDQEISINIQYLK 406
CsTPS30PK  ELFTNAVERWDISAMDGL-PEYMKTCFLALYNFINDLPFDVLKGEEGLHIIKFLQ 437
CsTPS5FN   SLFQHALGRWDINTIDQL-PDYMKIFFLATYNVVNEMAYDVLKQNGILI-IKYLK 388
CsTPS8FN   ELLTKAIQRWDINCMDKLEPEYLRTYYKVMLESYEEFEKELKKEELY--KLEYAK 368
CsTPS7FN   QLLTKAMQRWDINCMDKLEPEYLKTYYKVMLESYEEFEKELKKEELY--KLEYAK 368
CsTPS9FN   ELLTESINRWDLNCADQLRPECLQTFYKVLLNCYEEFESELGKEESY--KVYYAR 375
CsTPS4FN   KLLIEAIDRWDKNCMDKLHPEYLQKYYKILLQSFEEFEQEFEKEETY--KVYYGK 384

              500                       520                       540
CsTPS3FN   KSWVDLCKCYLQEAKWYYSGYQPTLEEYTELGWLSIGASVILMHAYFCFTN---- 493
CsTPS1FN   KLMVELCKHHLQEAKWFYSGYKPTLQEYVENGWLSVGGQVILMHAYFAFTN---- 491
CsTPS2FN   NSWAELCRCYLEEAKWFYSGYKPTLKKYIENASLSIGGQVIFVYAFFSLTK---- 429
CsTPS33PK  RTWVDFCKHQLQEAKWFHSGYKPTFEEYINNAWISVSGPIILMDAYFSLTN---- 480
CsTPS13PK  KTWVDMCRGFLQEAKWYYSGYTPTLEEYIENGWISVGAPVLIVHAYFSHANYNHT 463
CsTPS6FN   KTWVDMCKSFLQEAKWYYSGYTPTLEEYIENGWISVGAPVILVHAYFFHANNNRT 461
CsTPS30PK  KSWADLCKSYLREARWYYNGYTPSFEEYIENAWISISGPVILSHLYFFVVNPNKE 492
CsTPS5FN   KTWTDLCKCYMLEANWYHSGYTPSLEEYIKNGWISIAEPLILVNLYCLITN---- 439
CsTPS8FN   EEMKRIIRAYFEEARWLNEGYLPRLDEHLRVSYVSCGYVMLIATSYVGM--HDI- 420
CsTPS7FN   EEMKRIIRAYFEEARWLNEGYLPSFDEHLRVSYISSGYVLLIATSYVGM--DDI- 420
CsTPS9FN   EAMKRLLGAYFSEARWLHEGYFPSFDEHLKVSLISCGYTMMIVTSLIGMK--DC- 427
CsTPS4FN   ETFKRLLKGYFEEARWLNEGYMPSLEEHLKVSLVTSGYFMLMACSLVGMKSNNI- 438

                                   560                       580                       600
CsTPS3FN   -PITKQDLKSLQLQHHYPNIIKQACLITRLADDLGTSSDELNRGDVPKSIQCYMY 547
CsTPS1FN   -PVTKEALECLKDGH--PNIVRHASIILRLADDLGTLSDEMKRGDVPKSIQCYMH 543
CsTPS2FN   -SITNEALESLQEGHY--AACRQGSLMLRLADDLGTSSDELKRGDILKSVQCYMH 481
CsTPS33PK  -PVTKDAINLLELG--YPPIIYHASMILRLTDDLGTSNDEMKRGDIPKSIQCYMN 532
CsTPS13PK  VTSSKEIFECFEHG-YYPAIIRHSAIILRLTNDLATSSEELKRGDAPTSIQCYMQ 517
CsTPS6FN   ITNTKEIFECLEYG-YYPAIIRHSAIILRFTNDLATSSEELKRGDAPTSIQCYMQ 515
CsTPS30PK  NALLSTCFDG------YPTIIRHSSMILRLKDDMGTSTDELKRGDVPKSIQCKMY 541
CsTPS5FN   -PIKEDDIDCLLQ---YPTFIRISGIIARLVDDLGTSSDELKRGDNPKSIQCYMK 490
CsTPS8FN   --VTHETLDWLSKD---PKIISASTLLSRFMDDIGSRKFEQERNHIPSTVDCYMK 470
CsTPS7FN   --VTHETLNWLSKD---PKIVSASTLLSRFMDDIGSRKFEQERNHVLSTVECYMK 470
CsTPS9FN   --VTKQDFEWLSKD---PKIMRDCNILCRFMDDIVSHKFEQQRDHSPSTVESYMR 477
CsTPS4FN   --VTKQVFEWLSKD---PKIVRASASVCRYMDDVAGHKNEQERNHIPSTIECYMK 488

                     620                       640                       660
CsTPS3FN   DNNATEDE-AREHIKFLISETWKDMN------KKDED-ESCLS----------EN 584
CsTPS1FN   DTGASEDE-AREHIKYLISESWKEMN------NEDGNINSFFS----------NE 581
CsTPS2FN   ETGVSEDE-AREHIKFLISEIWKEMN------DEDE-YNSIFS----------KE 518
CsTPS33PK  DTGVSEDE-ARDHMKFLISELWKEIN------NEDENMDSPFS----------KQ 570
CsTPS13PK  EKNVSEEE-AREHIKFLINEAWKEMN------ND----VGLYP----------IS 551
CsTPS6FN   EKIVSEEE-AREHIKFLINEAWKEMN------ND----VGLYP----------IS 549
CsTPS30PK  EDGISEEE-ARQRIKLLISETWKLINKDYINLDDDDDGGDDYSPMFYKSNNINKA 595
CsTPS5FN   ENGICDEKNGREHIRNLISETWKEMNEARVG-------ESPFS----------QA 528
CsTPS8FN   QYGVSEEEAIKELNKRVVT-YWKEINEDFIRPTV-----------------VPFP 507
CsTPS7FN   QYEVSEEEAVKELNKRVAN-CWKEINEDFIRPTS-----------------VPFP 507
CsTPS9FN   QYGVSEQEACDELRKQVIN-SWKEINKAFLRPSN-----------------VPYP 514
CsTPS4FN   QYGVSEEEACDEMNRRVVI-AWKEINEEFLKPTE-----------------AASP 525
```

```
                             680                 700
                             |                   |
CsTPS3FN   FVEVCKNMARTALFIYE-NGDGHGSQ-NSLSKERISTLIITPINIPK* 630
CsTPS1FN   FVQVCQNLGRASQFIYQ-YGDGHASQ-NNLSKERVLGLIITPI--PM* 625
CsTPS2FN   FVQACKNLGRMSLFMYQ-HGDGHASQ-DSHSRKRISDLIINPI--PL* 562
CsTPS33PK  FLQNCKNLARISQFIYQ-YGDGHASQ-DSLSKQRISELIINHI--PS* 614
CsTPS13PK  LTEDATNFAKMGFFIYQ-HGDGHSSQ-DNQSKQKISSLIIEPIPLYT* 597
CsTPS6FN   LTEDATNFAKMGFFIYQ-HGDGHSSQ-DNQSKQKISSLIIEPIPLYT* 595
CsTPS30PK  FIEMCLNLGRMAHCIYQ-YGDGHGIQ-DRQTKDHVLSLLIHPIPLTQ* 641
CsTPS5FN   FIETAIDFVRTAMMIYQKEQDGVGTNFDHYTKDGIISLFFTSIPI*-- 574
CsTPS8FN   ILVRVLNFTKVIDLFYKEGDDQY-TRVGKALKESIDALLIDSI--PL* 552
CsTPS7FN   ILFRIINLTKTADFMYREGGDQY-THVGKMLKDSIAALLIDPI--PL* 552
CsTPS9FN   VLSLVLNFSRVMDLLYKDG-DGY-THIGKETKNSVVALLIDQI--P*- 557
CsTPS4FN   ILVRALNLARVMDLLYKNG-DNY-TQVGKVTKDSVAVLLIDPI--P*- 568
```
